# Tuning of Electron-Donating Metal–Organic Frameworks toward High-Performance Triboelectric Nanogenerators for Self-Powered Shear Sensing

**DOI:** 10.1021/acsami.5c22950

**Published:** 2026-03-02

**Authors:** Tianhuai Xu, Lorenzo Donà, Jin-Chong Tan

**Affiliations:** † Multifunctional Materials & Composites (MMC) Laboratory, Department of Engineering Science, 6396 University of Oxford, Parks Road, Oxford OX1 3PJ, U.K.; ‡ Department of Chemistry, NIS and INSTM Reference Centre, University of Turin, Torino 10125, Italy

**Keywords:** triboelectric nanogenerators, metal−organic frameworks, composite materials, chemical functionalization, shear force sensing

## Abstract

Triboelectric nanogenerators (TENGs), which convert mechanical energy into electrical signals, have emerged as apromising platform for self-powered motion sensing. However, the development of high-sensitivity TENG sensors remains limited by the availability of tunable and efficient tribo-positive materials, which are electron donors. In this work, we present a material design strategy based on the incorporation of electron-donating functionalized metal–organic framework (MOF) fillers into a polyurethane (PU) polymer matrix. Three functional groups (−CH_3_, −NH_2_, and −OH) were systematically studied to investigate their influence on triboelectric performance. The resulting composite membranes demonstrated tunable charge-donating behavior and improved electrical output, with the −OH-modified MOF yielding the highest electrical output of 197.6 ± 1.3 V and 0.47 ± 0.08 μA/cm^2^, which are 2.3 and 3.2 times higher than that of the pristine PU. The enhanced charge-donating mechanism was elucidated through a combination of advanced micro- and nanoscale chemical and mechanical analysis. Theoretical calculations employing *ab initio* density functional theory (DFT) were performed to reveal the electron distribution within the periodic MOF structure. Furthermore, the practical application of the optimized TENG device was demonstrated in a single-electrode shear sensor configuration, exhibiting high sensitivity in sliding motion detection. This study highlights a scalable and biocompatible strategy for improving tribo-positive materials and advancing the performance of tunable TENG-based sensors to enable shear force monitoring.

## Graphical abstract

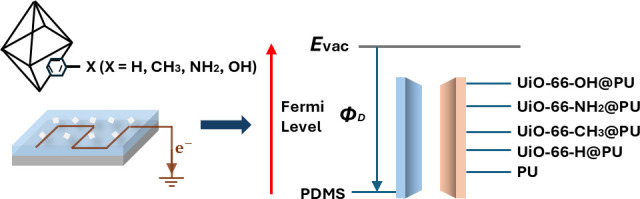

## Introduction

With the rapid advancement of artificial intelligence and the emergence of the Internet of Things (IoT) era, there is a growing demand for high-performance sensors capable of continuous and reliable data acquisition. Among emerging technologies, triboelectric nanogenerators (TENGs) have gained significant attention as promising alternatives to conventional sensors, owing to their self-powered operation and ease of fabrication.^1–4^ By coupling contact electrification with electrostatic induction, TENG-based sensors convert dynamic mechanical inputs into measurable electrical signals without the need for an external power source. Compared to traditional force sensors, TENG sensors offer distinct advantages, particularly in their high sensitivity to dynamic forces such as normal impacts and shear motion.^5,6^ Their versatile working modes and simple architectures have a great potential for integration into diverse applications, including soft robotics,^7,8^ wearable electronics,^9,10^ and biomedical implants.^11,12^

Despite these benefits, challenges remain that limit the broader adoption of TENG-based sensors. Low sensitivity has always been an obstacle, which is closely tied to the output performance of TENGs. Devices with higher electrical output generally exhibit improved sensitivity and broader detection ranges. To address this, various strategies have been pursued, with material development being the most prominent.^13–15^ Historically, efforts have almost exclusively focused on tribo-negative materials due to the availability and tunability of electronegative polymers. In contrast, the choice of tribo-positive materials is largely limited to metals (e.g., Cu and Al) and natural/synthetic polymers rich in amine or hydroxyl groups (e.g., nylon, cotton, and rabbit fur). These materials are often difficult to process either due to their relatively low mechanical stiffness or poor formability, which has significantly constrained the design space for tribo-positive layers.^16^ Additionally, many high-performance tribo-negative materials (such as fluorinated ethylene propylene (FEP), polyvinylidene fluoride (PVDF), and polytetrafluoroethylene (PTFE)) contain high levels of fluorine, raising environmental and health concerns, especially when used in wearable applications. These fluorinated polymers can pose threats to the environment after disposal and may cause adverse effects upon prolonged skin contact.^17^ Therefore, there is a pressing need to develop sustainable, biocompatible, and high-performance tribo-positive materials for next-generation TENG sensors.

One promising route to enhance triboelectric performance is the introduction of polar functional groups, which can facilitate charge separation at the interface. This has been achieved primarily through two approaches: surface modification and filler incorporation. Surface modification typically involves techniques such as radical injection,^18^ ion doping,^19^ plasma treatment,^20,21^ or the introduction of self-assembled monolayers (SAMs).^22,23^ While effective, these treatments are limited to the surface layer and are often unstable under mechanical wear or environmental exposure to ultraviolet (UV) light, heat and moisture.^24,25^ Durable devices with a long-term stability is central to practical applications.

On the other hand, the incorporation of functional fillers provides a more robust and sustainable means of achieving long-term performance enhancement. Metal–organic frameworks (MOFs), with their tunable chemical structures and physical versatility, have emerged as promising fillers for chemical doping.^26–29^ The first attempt was made in 2020 by Guo et al., where fluorinated KAUST-8 was introduced into a PDMS matrix, achieving an output of 530 V and 0.53 μA/cm^2^.^30^ Building on this work, Wang et al. incorporated highly fluorinated UiO–66–4F into PDMS and obtained a remarkably high output of 937.4 V and 3.4 μA/cm^2^.^31^ In 2023, Wen et al. functionalized UiO–66 with a series of groups and systematically studied their effects on the triboelectric output of PDMS composites. The performance followed the order −NO_2_ > −Br > −H > −NH_2_, consistent with the electron-withdrawing strength of the functional groups.^32^ More recently in 2025, Ye et al. embedded halogenated ZIF-8 derivatives into PVDF fibers, reporting a stable output of 312.4 V and 1.2 μA/cm^2^ for the ZIF-8-Cl@PVDF composite.^33^

Based on these findings, which demonstrate the benefits of electron-withdrawing groups for enhanced charge accumulation on tribo-negative materials, we hypothesize in contrast that electron-donating groups, prevalent in many tribo-positive materials, may similarly facilitate positive charge formation and improve sensor sensitivity. In this study, we select polyurethane (PU) as the tribo-positive polymer matrix due to its biocompatibility, mechanical durability, toughness, and inherent electron-donating nature, derived from its abundant urethane and ester linkages.^34–36^ UiO–66, as a biocompatible Zr-based MOF, is chosen as the parent MOF for functionalization due to its well-defined chemical structure and chemical tuning strategy.^37,38^ We synthesized isostructural UiO–66 derivatives and show that the electron-donating behavior of the composite membrane can be effectively modulated by tuning the functional groups in the ligand of the MOF fillers. The underlying mechanism behind the improved charge transfer was elucidated through experimental characterization of work function, supported by theoretical calculations employing *ab initio* density functional theory (DFT) that revealed the electronic structures within the UiO–66 derivatives. Finally, we demonstrate the practical utility of the resulting TENG device by validating its performance as a highly sensitive shear sensor.

## Methods

### Materials

All chemicals used in this study are commercially available. Sylgard 184 (PDMS elastomer and curing agent) was obtained from Dow Corning. Zirconium chloride and polyurethane were purchased from Sigma-Aldrich. Terephthalic acid (BDC = 1,4-benzenedicarboxylate) was purchased from Acros Organics. 2-Methylterephthalic acid, 2-hydroxyterephthalic acid were purchased from Fluorochem. 2-Aminoterephthalic acid was purchased from Thermo Fisher.

### Synthesis of UiO–66–X Nanoparticles

For UiO–66 synthesis, 233.04 mg of zirconium chloride (1 mmol) and 215.97 mg of terephthalic acid (1.3 mmol) were mixed together in 10 mL of DMF solution. Two mL of concentrated hydrochloric acid was added to the mixture and the solution was sealed in an autoclave and heated in an oven at 120 °C for 24 h. The product was washed with dimethylformamide (DMF) for 3 times and then once with methanol, after which it was activated in a vacuum oven overnight before use. The other UiO–66–X derivatives were synthesized in a similar fashion with the same molar amount of other modified BDC linkers.

### Fabrication of UiO–66–X@PU TENGs

The stock solution was prepared by dissolving 13.7 g of polyurethane pellets into 86.3 g of DMF solvent. All the synthesized UiO–66 aggregates were crushed into powders before blended into the polymer matrix. The ground particles were added into PU solution at a weight ratio of 2 wt % and were homogeneously dispersed using a homogenizer (IKA disperser) at 7500 rpm for 5 min. The mixture was then degassed in a vacuum glass chamber for 10 min to remove all the trapped air bubbles. To minimize the gap between the film and the electrode, the degassed mixture was casted directly onto an indium tin oxide (ITO)-coated polyethylene terephthalate (PET) substrate using a doctor-blade casting machine, after which it was cured in the oven at 60 °C for 5 h. The average thickness of the thin membrane of the nanocomposite films was found to be 63 ± 3 μm. Copper wires were directly attached to the ITO electrodes via Cu tapes. Rubber sheets were placed underneath the PET substrates as buffer layers for improved mechanical contact. The nominal contact area of the device was 3 × 3 cm^2^.

### Material Characterizations

The surface morphologies of the as-prepared MOF crystals were examined under a field emission scanning electron microscope (FESEM LYRA3 GM TESCAN). The X-ray diffraction (XRD) patterns of the MOF nanocrystals were obtained from a Rigaku MiniFlex diffractometer with a Cu Kα source (1.541 Å). Fourier transform infrared (FTIR) spectroscopy was performed with a Nicolet iS10 FTIR spectrometer equipped with an attenuated total reflectance (ATR) module. The synchrotron radiation far-IR (Terahertz) spectra of the MOF crystals were recorded on a Bruker Vertex 80v FTIR spectrophotometer equipped with an ATR accessory at beamline B22 in Diamond Light Source. The Raman spectra was obtained using a confocal Raman microscope (Bruker SENTERRA II) in B22. The surface height topography and the work function were characterized using the alternating current (AC) air tapping mode and the Kelvin probe force microscopy (KPFM) mode, respectively, on an atomic force microscope (Oxford Instruments Cypher-ES AFM). Near-field infrared nanospectroscopy (nano-FTIR) of the composite film was obtained from a scattering-type scanning near-field optical microscope (Neaspec s-SNOM), equipped with a conductive Pt-coated AFM tip operating under the tapping mode. Further details on nano-FTIR of MOF samples can be found in reference.^39^

### Contact-Separation-Mode TENG Output Measurements

All TENG measurements were performed on a customized contact-separation test rig, with the cyclic loading controlled by a power supply, a function generator, and a magnetic shaker (Figure S6). During a standard test, the prepared TENG devices were attached to a sample holder disc, and the amplitude of displacement was kept at 1.5 mm. The tapping frequency was 2 Hz and the tapping force was maintained at around 80 N determined from a load cell (Figure S7). The output voltage was recorded on an oscilloscope (Picoscope 5442D) with a 100 MΩ high voltage probe (Rigol RP1300H). The current output and charge transfer were measured employing an electrometer (Keithley 6514).

### Shear Sensing Test Procedure

To evaluate the shear sensing performance of the TENG devices, a simple manual sliding test was performed. Copper electrode segments were cut to dimensions of 0.5 cm × 2.5 cm, with an intersegment spacing of 1 cm. A PTFE plate, cut to match the size of the electrode segments, was manually slid across the surface of the shear TENG in a unidirectional motion. During all measurements, the electrodes were grounded, and the generated signals were recorded using the same oscilloscope. Although precise control over the sliding speed and applied normal force was not available, consistent motion was maintained across repeated trials to ensure comparability. For car speed detection study, a larger shear sensor was used, with electrode dimensions of 0.8 cm × 9.8 cm and a spacing of 2 cm. A small model car was manually slid across the surface of the composite membrane at varying speeds, and the output signals were recorded.

### Density Functional Theory (DFT) Calculations

To investigate the electronic properties of the functionalized UiO–66–X derivatives, periodic DFT calculations were performed at the PBEsol0–3c level of theory,^40,41^ as implemented in the CRYSTAL23 code.^42^ A (75, 974) pruned grid was employed for the numerical evaluation of the Exchange-Correlation term, corresponding to the XLGRID keyword as used by the CRYSTAL code. Default convergence criteria for geometry optimization were employed, while the tolerances for one- and two-electron integrals calculation were set to 10^–7^, 10^–7^ for the Coulomb and to 10^–7^, 10^–7^, 10^–25^ for the Exchange series, respectively. The shrinking factors for the diagonalization of the Kohn–Sham matrix in the reciprocal space were set to 2 for the Monkhorst–Pack net and to 2 for the Gilat net. A full relaxation of both unit cell parameters and atomic positions was performed. On the optimized geometries, vibrational frequencies at the Γ point were computed using two-point numerical differentiation, and the Berry phase approach was employed to calculate the IR intensities. Subsequently, IR spectra were simulated by a Lorentzian peak broadening with a full width at half-maximum (FWHM) of 10 cm^–1^ and the predicted harmonic frequencies were scaled by a factor of 0.96 to account for anharmonicity effects. The Jmol software was used to plot both crystalline orbitals (CO) and electrostatic potential maps (EPM).^43^

## Results and Discussion

### Material Characterizations

The UiO–66 derivatives, thereafter designated as UiO–66–X, were synthesized using identical reaction times and metal-to-linker ratios, with varying functional groups (where X = −H, −CH_3_, −NH_2_, and −OH), as illustrated in Figure 1a. The morphology of the as-synthesized UiO–66–X nanocrystals, imaged by FESEM, is shown in Figure 1b. All samples exhibit a similar crystal morphology and comparable crystal size. The average particle size of the UiO–66–OH crystals is approximately 255 ± 9 nm, based on grain size distribution analysis of an AFM image (Figure S1). A representative AFM cross-sectional image of a single nanocrystal reveals an octahedral geometry with an aspect ratio of around 1.2 (Figure 1c).

**Figure 1:**
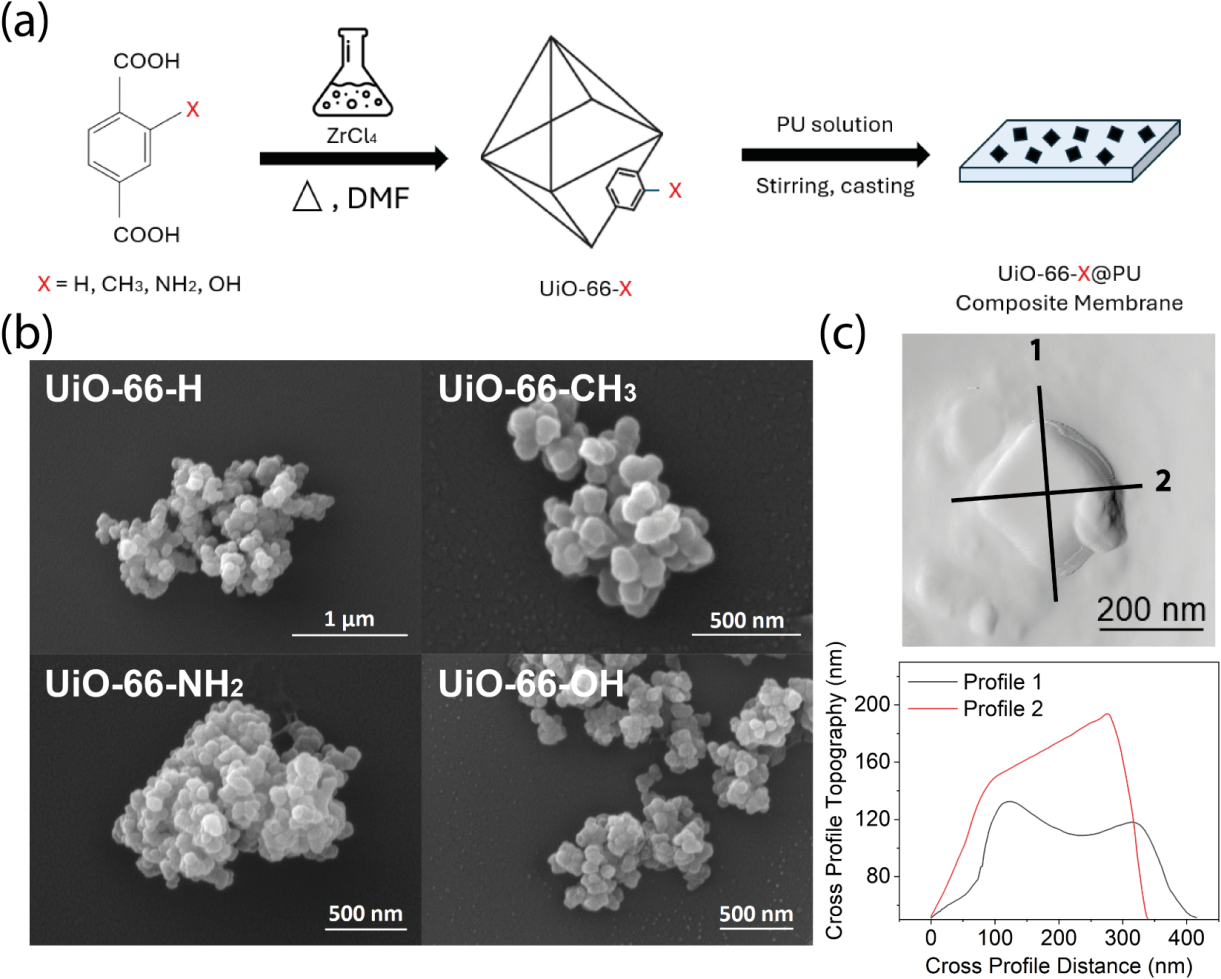
(a) Schematics illustrating the sample preparation steps for different UiO–66–X derivatives (where X = H, CH_3_, NH_2_, OH) and the resultant PU composite films incorporating UiO–66–X nanoparticles. (b) FESEM micrographs of the as-synthesized UiO–66–X nanocrystals. (c) AFM height topography of a UiO–66–OH single crystal and the corresponding cross-section profiles along two orthogonal axes.

The powder X-ray diffraction (PXRD) patterns of the UiO–66 derivatives are presented in Figure 2a. All samples exhibit prominent diffraction peaks corresponding to the (111) and (200) planes, which are consistent with the previously reported patterns for UiO–66.^44^ The close match between the PXRD profiles of the functionalized derivatives and that of the parent MOF indicates that the introduction of functional groups does not alter the overall crystallinity or the framework structure.

**Figure 2:**
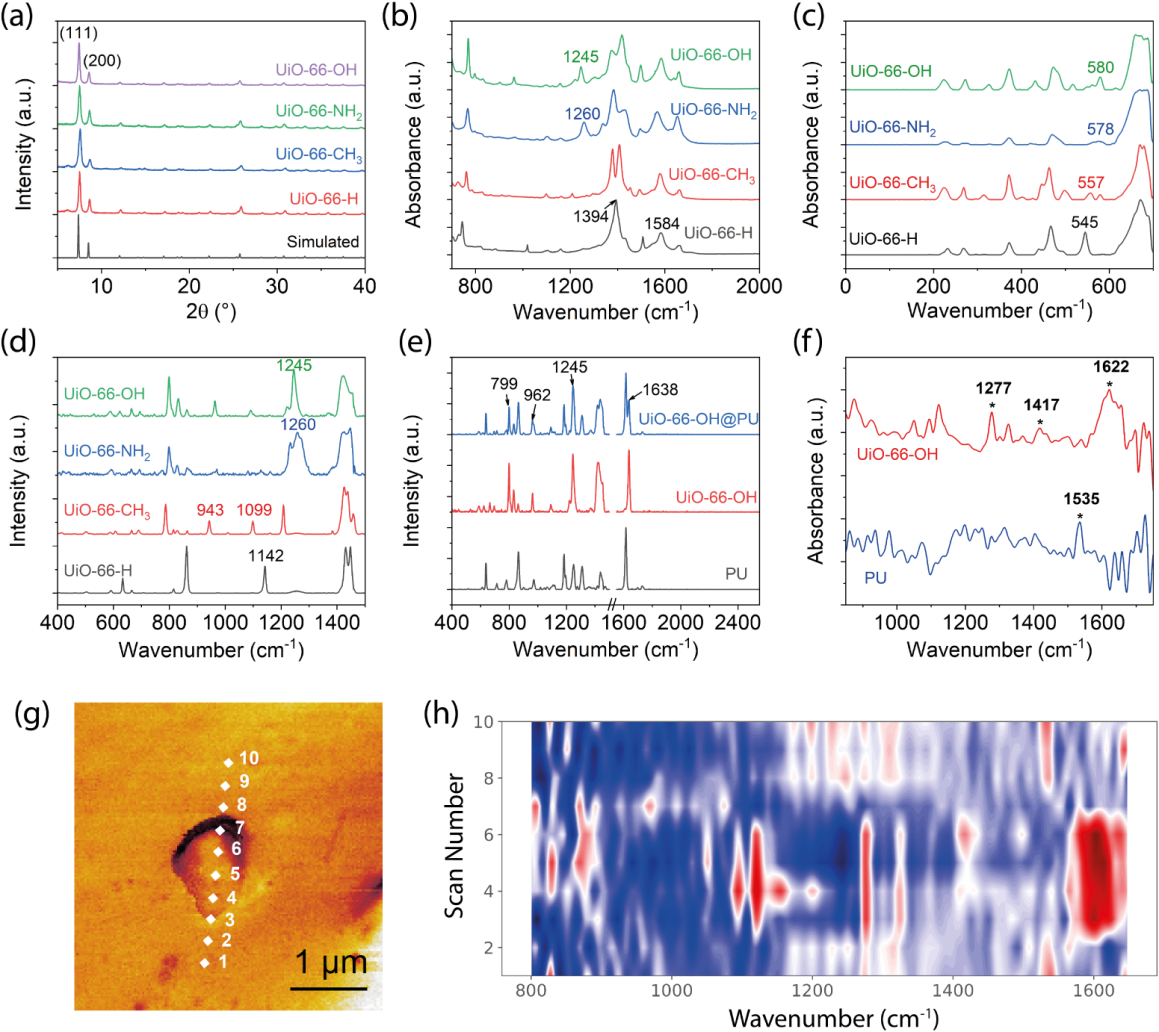
(a) XRD patterns of the as-synthesized nanoparticles of UiO–66–X compared with simulated pattern for UiO–66 (generated from crystallographic information file (CIF) in the Cambridge Structural Database, CCDC code: RUBTAK). (b) ATR-FTIR spectra of the UiO–66–X nanoparticles. (c) Synchrotron far-IR (THz) spectra of the UiO–66–X nanoparticles. (d) Raman spectra of the UiO–66–X nanoparticles. (e) Raman spectra of the pristine PU, the as-synthesized UiO–66–OH nanoparticles and the UiO–66–OH@PU composite film. (f) Near-field nano-FTIR spectra of the UiO–66–OH nanocrystal and PU. (g–h) White light near-field infrared image of a UiO–66–OH agglomeration on the PU composite film and nanoFTIR contours showing the corresponding line scans across an agglomeration present on the composite film surface.

The Fourier-transform infrared (FTIR) spectra confirm successful synthesis of the UiO–66 and its derivatives, as shown in Figure 2b. The characteristic peaks for the parent MOF are identified at 1394 cm^–1^ and 1584 cm^–1^, which correspond respectively to the symmetric and asymmetric stretching of the carboxylate (BDC) groups. When −CH_3_ group is attached, the 1394 cm^–1^ peak is split due to the asymmetric perturbation of the carboxylate vibrations from the methyl functional group. Substitution with −NH_2_ group results in an additional peak at 1260 cm^–1^, which is assigned to C–N bond stretching. Functionalization of the −OH group leads to an extra peak at 1245 cm^–1^, which is related to the bond stretching in the C–O bond. The far-IR spectra obtained using a synchrotron radiation source shown in Figure 2c reveal additional information about the metal–ligand interactions and collective modes in the terahertz region. The slight shift and dampening of the IR band at 545 cm^–1^ suggest that functionalization hinders the symmetric stretching of the Zr–O coordination bond. The FTIR peaks in both the mid-IR and far-IR domains align well with the IR bands predicted by DFT, as compared in Figures S2 and S3. The FTIR bands confirm the presence of electron-donating moieties in the MOF structures responsible for charge separation.

The Raman spectra of the UiO–66–X nanoparticles are presented in Figure 2d. A characteristic peak is observed at 1142 cm^–1^ for UiO–66, which is attributed to the CH in-plane bending vibration of the aromatic ring. Upon functionalization with methyl group, this peak exhibits a red shift, indicating a reduction in C–H bond strength due to the electron-donating nature of the substituents. A new Raman mode also emerges at 943 cm^–1^, which is assigned to the C–H out-of-plane bending induced by the methyl substituent. In addition, the incorporation of amine and hydroxyl groups introduces new Raman bands at 1260 cm^–1^ and 1245 cm^–1^, corresponding to the stretching vibrations of the C–N and C–O bonds, respectively. The Raman spectra for the UiO–66–OH@PU composite membranes are compared in Figure 2e. The composite membrane retains all the characteristic Raman bands for the pristine PU film, with additional peaks showing up at 799, 962, 1245, and 1638 cm^–1^, which are all characteristic Raman peaks observed for UiO–66–OH.

The UiO–66–OH-loaded PU films were further characterized using nano-FTIR spectroscopy, which enables spatial resolution down to 20 nm.^39^ As shown in Figure 2f, the UiO–66–OH aggregates and the PU polymer matrix can be readily distinguished based on their nano-FTIR spectral signatures. The spectra obtained from both the MOF aggregates and the surrounding polymer matrix show good agreement with the corresponding bulk FTIR spectra (Figure S4). The slight shifts observed in the nano-FTIR peaks of the UiO–66–OH crystals are likely due to enhanced near-field interactions associated with their highly ordered crystalline structure.^45^ Figure 2g presents a line scan across the polymer surface that intersects a UiO–66–OH agglomerate. The corresponding heatmap in Figure 2h reveals a smooth spectral transition from the PU matrix to the UiO–66–OH domain, with characteristic peaks at 1277, 1417, and 1622 cm^–1^ for UiO–66–OH, and a prominent peak at 1535 cm^–1^ corresponding to the C–N stretching vibration in the urethane linkage of PU. The absence of significant spectral overlap or peak shifts suggests minimal chemical interaction between the MOF crystals and the polymer matrix, indicating that MOF incorporation does not substantially alter the chemical environment of the PU substrate.

### TENG Performance

To evaluate the charge-donating capability of the nanocomposite membranes of UiO–66–X@PU, their triboelectric outputs were measured in a contact-separation mode, as illustrated in Figure 3a. Polydimethylsiloxane (PDMS) was employed as the tribo-negative layer due to its excellent electron-withdrawing properties.^46^ Upon contact, charges of opposite polarity accumulate on the surfaces of the dielectric films, inducing an electric potential difference across the back electrodes. The relative motion between the two layers generates alternating positive and negative peaks in the output signal. A MOF filler loading scaling test was performed to determine the effect of filler wt % content. It was determined that 2 wt % was the optimal loading amount, as shown in Figure S8. From the micrographs shown in Figure S9, at lower loading amount, the particles are uniformly dispersed within the polymer matrix. Upon further loading, agglomeration of nanoparticles dominates, thereby compromising the effective contact area between the contact surfaces. The open-circuit voltage, short-circuit current density, and transferred charge during each cycle are presented in Figure 3b–d. Compared to pristine PU, the addition of parent UiO–66 fillers led to increases in average open-circuit voltage, short-circuit current density, and transferred charge density (all averaged from 20 working cycles) from 84.4 ± 0.5 to 133.2 ± 0.7 V, 0.14 ± 0.01 to 0.30 ± 0.06 μA/cm^2^, and 0.74 ± 0.03 to 1.08 ± 0.02 nC/cm^2^, respectively. This moderate enhancement is attributed to increased surface roughness and improved charge trapping capacity resulting from the porous crystalline structure of the MOF.^47^ Functionalization of UiO–66 crystals with −CH_3_ groups further enhanced the triboelectric output, resulting in an open-circuit voltage of 172.7 ± 1.3 V, a short-circuit current density of 0.33 ± 0.07 μA/cm^2^ , and a transferred charge density of 1.15 ± 0.02 nC/cm^2^. Although the methyl group is relatively neutral in terms of electron affinity, its incorporation led to a reduction in the average pore size within the framework. This structural modification contributed to an increased dielectric constant and enhanced polarizability, thereby improving the charge generation and storage capacity of the composite membrane.^48^ Substitution with electron-donating groups (−NH_2_ and −OH) led to further enhancement in triboelectric performance, with UiO–66–OH@PU exhibiting the highest output among all samples. The composite film achieved an open-circuit voltage of 197.6 ± 1.3 V, a short-circuit current density of 0.47 ± 0.08 μA/cm^2^, and a transferred charge density of 2.05 ± 0.03 nC/cm^2^, representing a 2.3-fold, 3.2-fold, and 2.8-fold rise in the TENG output, respectively, compared to the pristine PU-based TENG. Table S1 compares the maximum transferred charge density per unit stress. The UiO–66–OH@PU TENG reached the highest value of 230.5 μC m^–2^ MPa^–1^, which shows a ∼2.8 times better charge generation capacity compared with its pristine counterpart. The output trend is consistent between batches, as shown in Figure S10. To assess the capability of the UiO–66–OH@PU TENG device as a power source, its voltage output was measured across a range of external load resistances. As shown in Figure 3e, the output voltage increases sharply when the load resistance exceeds 1 MΩ and begins to plateau beyond 50 MΩ. Applying the equation P = V^2^/R, the device achieves a maximum peak power density of about 833 mW/m^2^ at an optimal load resistance of 10 MΩ.

**Figure 3:**
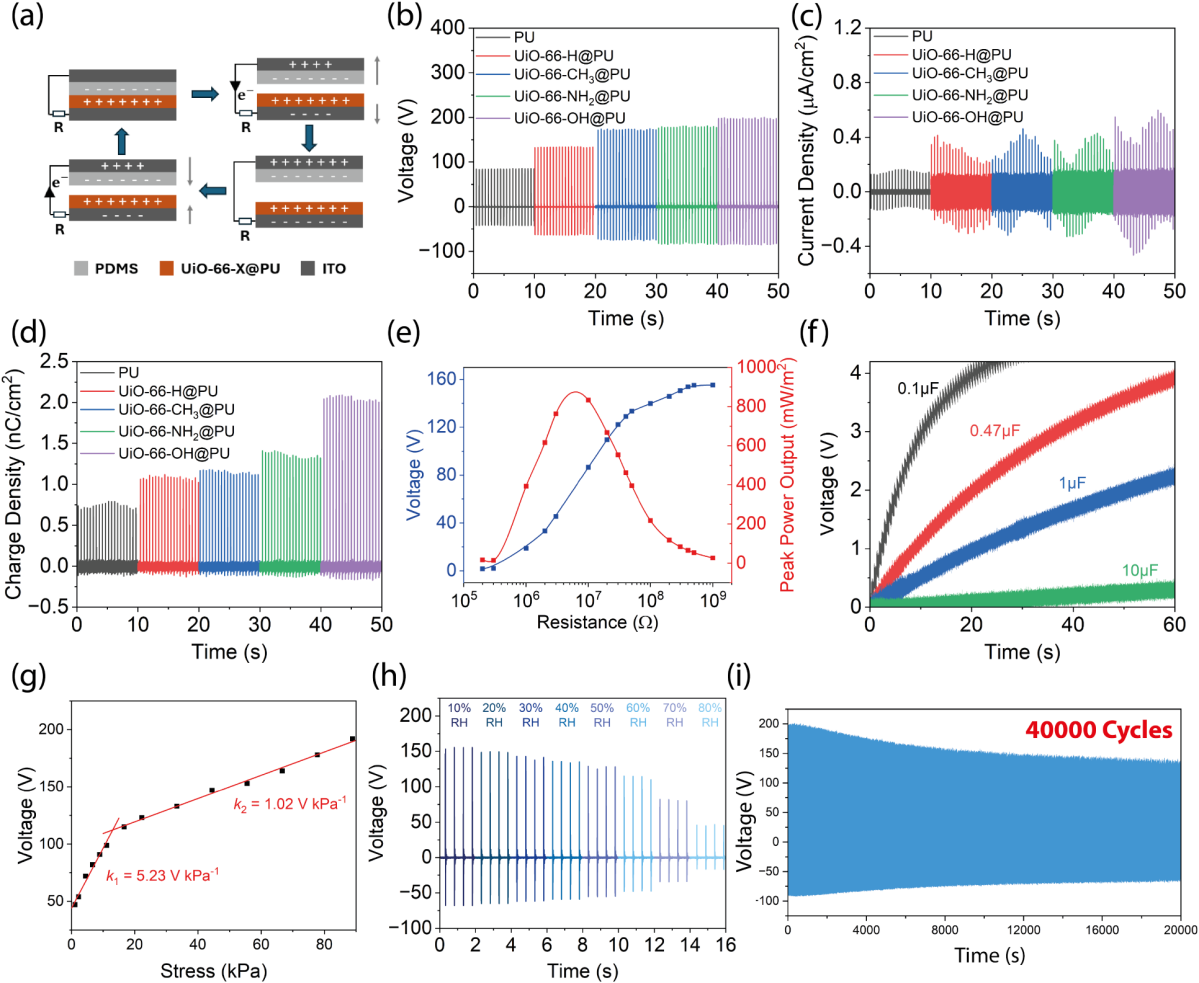
(a) Schematic illustrating the working principle of a dielectric-to-dielectric contact-separation mode TENG. (b–d) The open-circuit voltage, short-circuit current density, and charge transfer of the UiO–66–X@PU TENG devices. (e) The peak voltage output and its corresponding power density of the UiO–66–OH@PU TENG measured as a function of load resistance. (f) Voltage profiles for charging different capacitors by the UiO–66–OH@PU TENG operating at 2 Hz. (g) Voltage output of UiO–66–OH@PU TENG under varying stresses. (h) Voltage output of UiO–66–OH@PU TENG determined at a rising relative humidity level ranging from 10 to 80%RH. (i) Long-term durability of the UiO–66–OH@PU TENG measured over 40,000 contact-separation cycles, operating at 80 N impact load and 2 Hz tapping frequency.

To investigate the functionality of the TENG devices for practical use, they were integrated into a closed circuit to charge various capacitors. The charging performance of a 0.1 μF capacitor was compared across different composite membranes. Among them, the UiO–66–OH@PU TENG demonstrated the fastest charging rate, reaching 4.9 V within 50 s, as shown in Figure S11. Using the equation E = 1/2 CV^2^, we found that the total energy stored in a UiO–66–OH@PU TENG after 50 s is 7.2 times higher than that in a pristine PU TENG. Figure 3f presents the charging curves of the UiO–66–OH@PU TENG device when charging capacitors with capacitances of 0.1, 0.47, 1, and 10 μF. As the capacitance went up, the charging rate of the TENG device also decreased. The output of the TENG device under varying mechanical stress is compared in Figure 3g. The TENG device is more sensitive to increased forces when stress is low, reaching a pressure sensitivity of 5.23 V/kPa, which is more than 5 times higher than that at higher stresses. The performance of the TENG device under varying relative humidity (RH) levels was compared in Figure 3h. The open–circuit voltage remained relatively stable up to approximately 40% RH, but exhibited a sharp decline beyond this point, dropping to just 46 V at 80% RH. This reduction in output is attributed to the hydrophilic nature of the −OH groups. The incorporation of the UiO–66–OH fillers significantly reduced the hydrophobicity of the PU membrane, as evidenced by the decline in the water contact angle shown in Figure S12. The presence of these polar functional groups renders the composite film more susceptible to moisture, thereby compromising its humidity resistance and leading to a substantial loss in electrical performance.^49^ The durability of the TENG device over 40,000 working cycles were also recorded in Figure 3i. After 40000 cycles, the output voltage fell from 194 to 138 V, which corresponds to a 29% decrease. This drop in voltage may be attributed to dust accumulation on the contacting interface or change in mechanical properties due to material evolution (e.g., filler detachment, surface wear or polymer chain relaxation) after long contact duration.^46,50^

### Functionalization Effects

The better electron donating nature of the UiO–66–OH loaded PU film was further verified through Kelvin probe force microscopy (KPFM), which measures the contact potential difference between the sample surface and the Kelvin probe. The work function of the measured sample is directly correlated with the contact potential difference (V_CPD_) by the following equation:

$$V_{\mathrm{CPD}}=\frac{\phi_{\mathrm{tip}}-\phi_{\mathrm{sample}}}{-e}$$

where Ø_tip_ and Ø_sample_ are the work functions of the probe and the sample, respectively, and e is the charge of an electron.

The work function of a sample quantifies the minimum thermodynamic energy required to remove an electron from a solid surface and serves as a useful indicator of the electron affinity of the material. The contact potential differences between the tip and the composite membranes are mapped in Figure 4a, presented on a uniform color scale. Compared to previously reported value for PDMS (−2.25 V), all the PU composites exhibit much higher contact potential differences, which suggest effective charge separation between the contact materials.^46^ Compared to UiO–66@PU, when methyl functional group is attached, the average contact potential difference increased from 0.98 to 1.03 V. Functionalization of amine group and hydroxyl group significantly increased the average contact potential to 1.72 and 1.85 V, respectively, corresponding to a reduction of 0.74 and 0.87 eV in the work function. This trend is in strong agreement with the observed triboelectric output in Figure 3. According to the surface state model, when two materials come into contact, electrons tend to transfer from the material with a higher surface state to the one with a lower surface state.^51^ The observed decrease in the work function on the electron-donating side increases the energy gap between the contacting materials, thereby enhancing charge transfer, as indicated by the higher charge equilibrium state shown in the schematic in Figure 4b.

**Figure 4:**
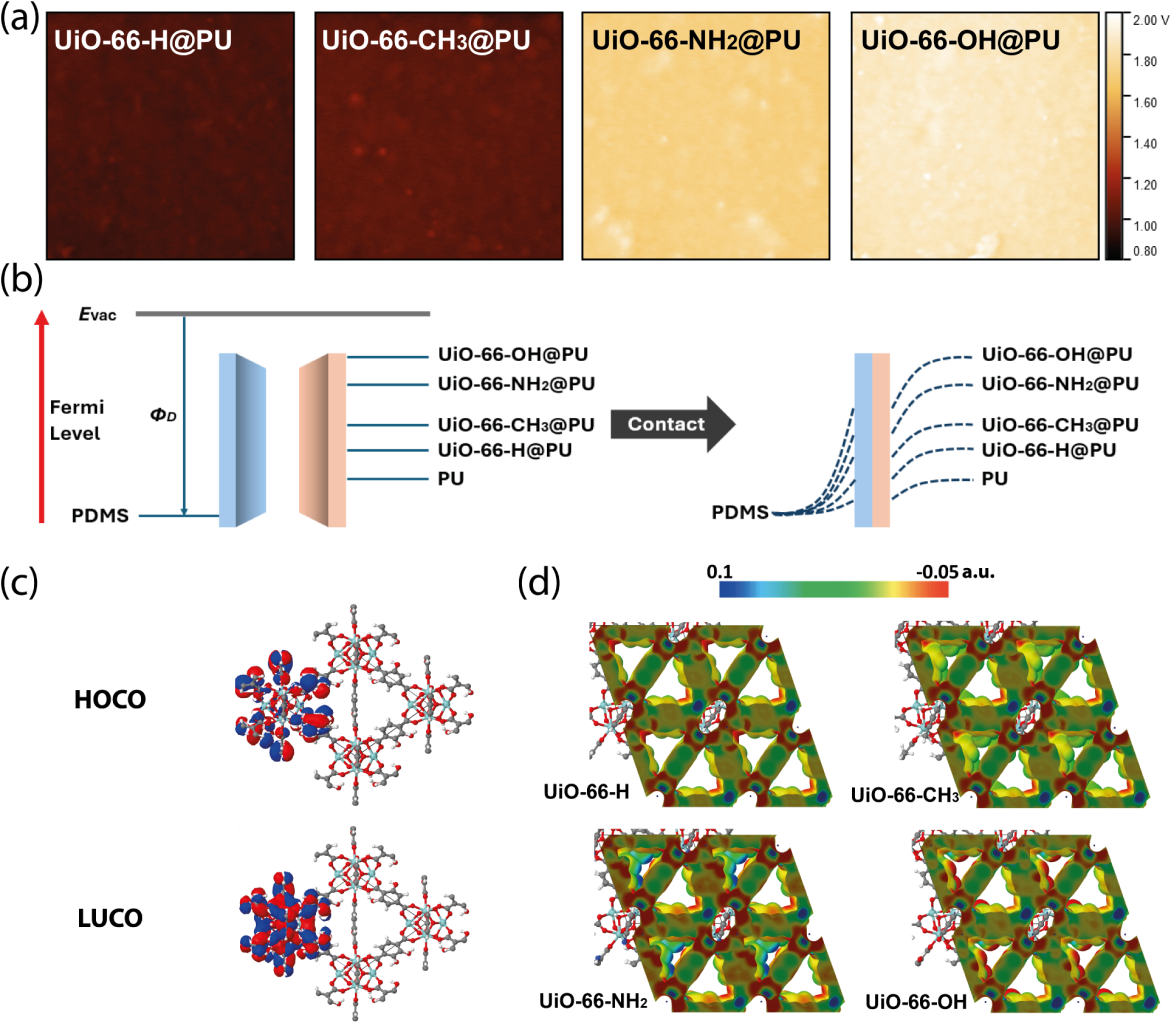
(a) Surface potentials of UiO–66–X@PU composite films from KPFM measurements, presented under the same color scale. (b) Surface state model elucidating the charge transfer process during contact electrification in the case of a dielectric–dielectric pair. Blue lines: fermi level of different dielectrics. E_vac_: vacuum energy level. Φ_D_: work function of the dielectric material from E_vac_ level. (c) The HOCO and LUCO crystal orbitals of a UiO–66–OH unit cell predicted by periodic DFT calculations. (d) Electrostatic potential maps calculated by DFT of the four different UiO–66–X frameworks. Red, green, and blue regions represent negative, zero, and positive potential values, in atomic units, respectively.

To support the proposed electron-donating mechanism, the electron distributions within the UiO–66 with various functional ligands were simulated using periodic DFT calculations. As shown in Figure 4c, the highest occupied crystalline orbital (HOCO) and lowest unoccupied crystalline orbital (LUCO) were visualized on the framework structures. Notably, in the structure of UiO–66–OH, the HOCO is primarily localized on the hydroxyl group, indicating that this functional group serves as an active site for charge transfer. Figure 4d presents the electrostatic potential maps of the four UiO–66 derivatives. Compared to the unmodified UiO–66, the nonpolar methyl group has almost no effect on the electron distribution within the molecule. However, functionalization with polar groups such as −NH_2_ and −OH results in more distinct regions of positive and negative electrostatic potential. Between them, the −OH group exhibits more negative potential, attributed to the high electronegativity of oxygen. Furthermore, hydrogen bonding between the carboxylate oxygen and the hydrogen atom of the hydroxyl group leads to partial charge accumulation on the oxygen atoms, further lowering the electrostatic potential along the hydroxyl-functionalized linkers.^52^ This intramolecular interaction also pulls electron density away from the carbonyl group, which weakens its suppression effect on the delocalization of π-electrons on the benzene ring in the BDC linker, increasing the transferrable charge on the ligand and restoring its electron-donating nature.^53^ The enhanced electron-donating ability is further supported by band-structure and density-of-states analyses (Figures S14 and S15), which show a shift of the electron density toward higher Fermi levels upon incorporation of electron-donating functional groups, thereby promoting charge donation.^54^ The electron-donating MOFs induce local interfacial dipoles and reach Fermi-level equilibrium with the surrounding polymer matrix, which effectively reduces the surface-level electron affinity of the composite.

The induced polarity shift is further confirmed by dielectric measurements. Figure S16 shows the dielectric constant (ε_d_) of the composite films across different frequencies. At a frequency of 1 MHz, compared to neat PU (ε_d_ ∼ 2.0), the more polar functional groups, namely −NH_2_ and −OH render the films more polarizable due to more dipoles formation, thus reaching a dielectric constant value of 3.6 and 4.3, respectively. A higher dielectric constant contributes to more effective capacitance of the TENG device, enabling higher surface charge density under identical contact conditions.^55^

In addition to electrical properties, the effect of functionalization on the adhesion of the composite membranes were evaluated using a pull-off test. The detailed experimental procedure and corresponding results are provided in Figures S17 and S18. A summary of the measured pull-off stresses and work of adhesion is presented in Table 1. The results show that functionalization with amine and hydroxyl groups increases surface adhesion, as evidenced by higher pull-off stress and work of adhesion values. This enhancement in adhesive properties promotes more efficient charge transfer during contact, as higher adhesion force enables more prolonged contact, and thus leading to a better chance for a more conformal contact. Furthermore, increased work of adhesion boosts the likelihood of material transfer between the contacting materials, when the adhesive energy exceeds the energy required for bond cleavage.^56^

**Table 1:** Comparison of the Pull-Off Stress and Work of Adhesion of Pristine PU and UiO–66–X@PU Film Samples^a^

Sample	Pull-off stress (MPa)	Work of Adhesion (pJ)
Pristine PU	0.268 ± 0.035	8.91 ± 0.86
UiO–66–H@PU	0.260 ± 0.063	7.80 ± 1.48
UiO–66–CH_3_@PU	0.259 ± 0.043	7.32 ± 0.95
UiO–66–NH_2_@PU	0.275 ± 0.054	9.24 ± 1.87
UiO–66–OH@PU	0.374 ± 0.046	11.53 ± 1.03

a Pull-off stress values were averaged from 8 measurements. Work of adhesion values were computed by Integrating the area found under the load-depth curves. The average and standard deviation values were derived from 5 separate measurements.

### Application of the TENG Device as a Shear Motion Sensor

The TENG device was integrated into a compact shear sensor for directional motion sensing. The working principle of the shear sensor is depicted in Figure 5a. As an object moves across the surface of the composite membrane, triboelectric charges induced on the surface generate a potential difference between the electrode and the ground. This potential difference produces a detectable signal each time the object passes over an individual electrode segment.

**Figure 5:**
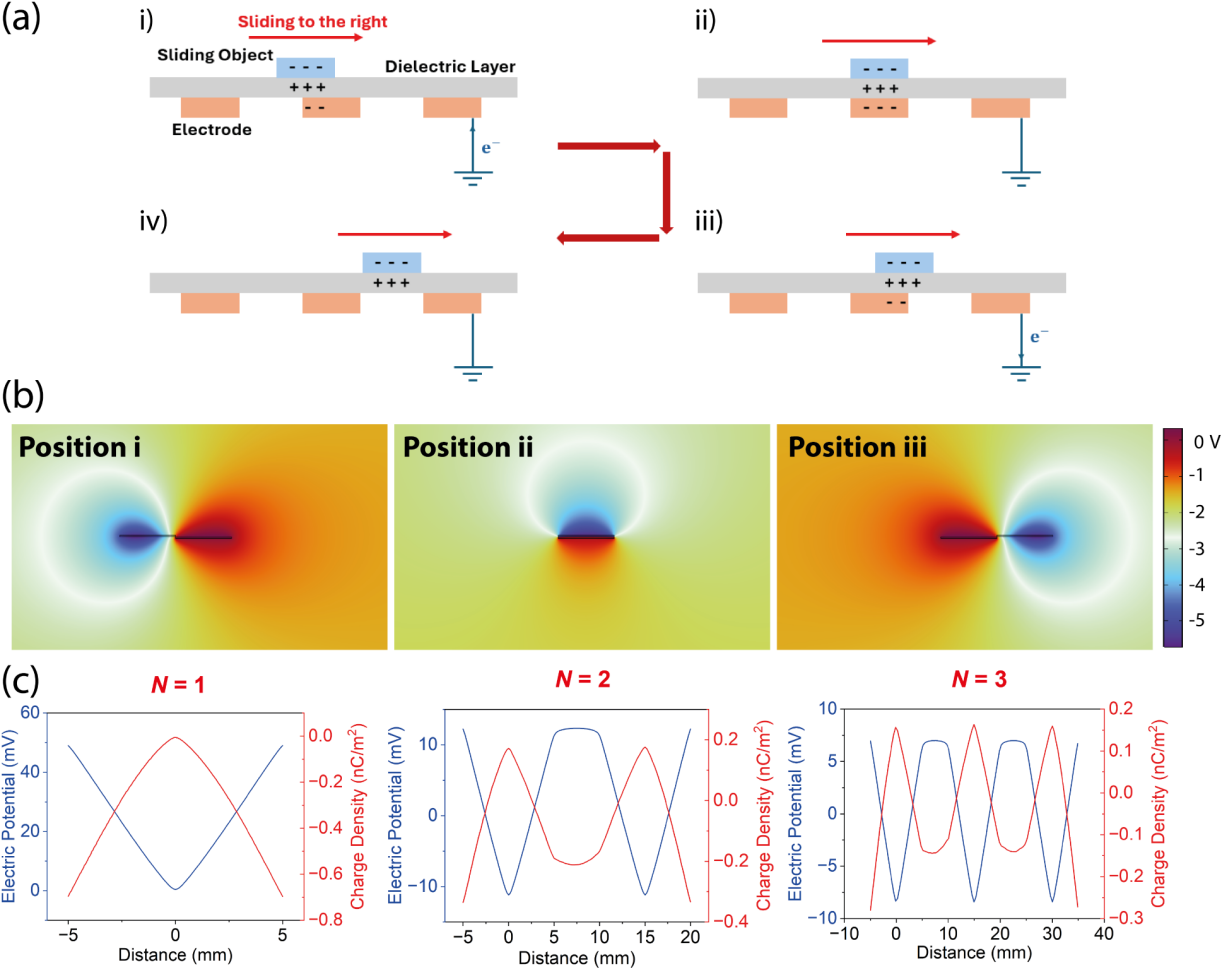
(a) Working principle of a single-electrode TENG under the sliding mode. (b) COMSOL finite-element (FE) modeling of the electric potential distribution for a sliding TENG with N = 1 segment. (c) FE simulated electric potential and charge density profiles of the TENG device at different sliding displacements, when N = 1, 2, and 3 segments.

To theoretically investigate the output behavior of introducing this interdigitated electrode configuration into a single electrode mode TENG device, we employed the finite element method (FEM) to simulate the voltage and the transferred charge density of electrodes with different numbers of segments using COMSOL software. The FEM simulation covers the entire unidirectional sliding event that starts off from the position where the far-end of the sliding object (PTFE) lines up with the near-end of the first electrode segment to the end position where the near-end of the sliding object touches the far-end of the last electrode segment.

Taken each charge transfer step as a unit step, for each sliding event, there will be a total of 2N unit steps for a sliding TENG with N segments. Initially, a uniformly distributed triboelectric surface charge density of 25 nC/m^2^ (determined to fit experimental data) was assigned on both contacting surfaces: negative charge on the PTFE layer and positive charge on the composite membrane. One unit step brings the sliding object to overlap one electrode segment, leading to a drop in electric potential, as shown in Figure 5b. The induced potential drop drives electrons to flow from the ground to the electrode, causing the transferred charge density to increase in this step. Vice versa, as the sliding object moves from the overlapping region to the other side of the electrode, electric potential rises again, and the transferred charge density returns to its original level.

Figure 5c illustrates the relationship between the number of electrode segments and both the electric potential and the transferred charge density. As the number of electrode segments increases, the number of signal peaks increases proportionally. Specifically, N electrode segments produce N pairs of sequential positive and negative peaks. Each peak is centered within its corresponding electrode segment. However, increasing the number of segments results in a reduction in the voltage across each individual electrode. This is attributed to a decrease in surface charge density, as the total charge is distributed over a larger surface area.

The shear sensing capability of the UiO–66–OH@PU TENG was further verified in experiments. A single-electrode mode TENG was adopted for structural simplicity, as illustrated in Figure 6a. The sensor comprises three layers: a composite membrane (UiO–66–OH@PU) on the top, an insulating layer (PET) at the bottom, and an interdigitated electrode positioned in between the sandwich structure. The electrode design adopts a serpentine configuration, wherein the electrode segments follow a continuous “S”-shaped sensor geometry. This electrode configuration ensures mechanical flexibility and deformability, making it well-suited for integration into wearable devices. The shear response of a one-segment electrode TENG using pristine PU and UiO–66–OH@PU is compared in Figure 6b. The peak-to-peak voltage increased from 72 mV to 142 mV, which indicates that the addition of fillers effectively enhanced the sensitivity of the shear sensor, while maintaining the mechanical compliance of the membrane, as confirmed from mechanical properties shown in Figure S19. Figure S21 also demonstrates that the shear sensor is quite sensitive to shear rate and normal load, proving its capability for the detection of subtle shear motion.

**Figure 6:**
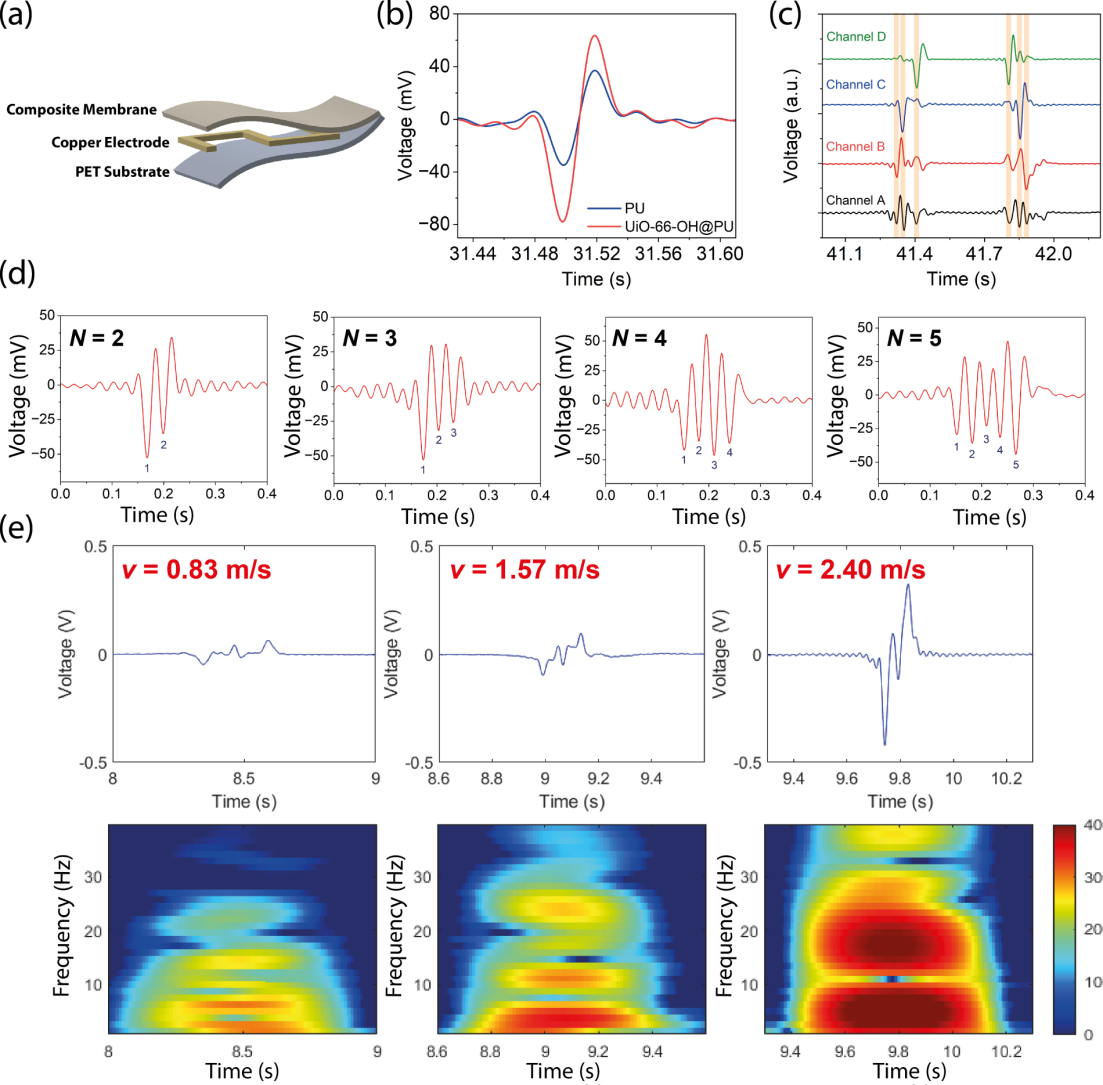
(a) Schematic showing the 3D layered structure of the TENG shear sensor. (b) Comparison of the signal output for the shear sensor constructed from the pristine PU polymer and the UiO–66–OH@PU composite film. (c) Comparison of the signal outputs of a 3-segment shear sensor under the serial (Channel A) and parallel (Channel B, C and D) configurations. (d) Relationship between the output signal and number of segments (N) used in the shear sensor. (e) Comparison of the signal output and the STFT processed output of a model car passing through the three-segment shear sensor.

To evaluate the feasibility of using a single-channel output as a substitute for multichannel acquisition, the output signals from three electrode segments were compared under both series and parallel configurations. Figure 6c shows that the peak positions in the single-channel signal align closely with those in the multichannel outputs. The sequence of the appearing signals from the single channels gives additional information about the direction of sliding, which is not directly manifested in the multichannel output. Other than the predominant peaks identified when sliding occurs above each electrode segment, minor peaks were observed for the single channel signals due to crosstalk and interference between electrode segments. Furthermore, the superimposed signal obtained from the single channel nearly overlaps with the combined signal from the three individual channels, as illustrated in Figure S23. The results demonstrate that the single-channel configuration effectively captures the key information embedded in the multichannel data and can be used to reconstruct or decode the individual channel outputs.

Figure 6d presents the experimental signal output after applying fast Fourier transform (FFT) filtering at 40 Hz to eliminate noise from the external power supply operating at 50 Hz. The potential difference between the electrode and the ground was recorded for each electrode configuration. The experimental results show good agreement with the simulation data, with the number of peaks corresponding to the number of electrodes used in the configuration. It should be noted that the simulation was performed in stationary mode and does not account for time-dependent effects. As a result, the simulated output reflects the spatial distribution of potential at discrete positions, whereas the experimental data captures the temporal evolution of the signal. The experimental results not only confirm the potential profile but also reveal additional information captured by the TENG-based shear sensor about the movement speed of the object concerned.

To evaluate its practical applicability, the shear sensor was used as a car speed detector for motion sensing. The filtered signal output is shown in Figure 6e. The average speed of the car was calculated by dividing the distance between the front and rear wheels by the time interval between sequential negative peaks in the signal. To further analyze the signal characteristics, short-time Fourier transform (STFT), which is suitable for processing nonstationary and transient signals, was applied to extract the frequency content of the signal over time.^57^ The results show that the motion signals intensify and shift toward higher frequencies as the car moves faster. Additionally, the broad frequency bands observed suggest that the car undergoes acceleration or deceleration while passing over the sensor. Notably, the low frequency components also become more pronounced at higher speeds. This is likely due to increased friction between the wheels and the surface of the sensor device, which generates stronger low-frequency oscillations. These findings demonstrate that the device can capture not only the speed of the sliding object but also its dynamic changes in motion behavior.

## Conclusions

In this work, we demonstrated an effective strategy to enhance tribo-positive materials by incorporating MOF fillers functionalized with electron-donating groups. Functionalized ligands were introduced during MOF synthesis, yielding isostructural UiO–66–X crystals with different terminal groups (where X = −H, −CH_3_, −NH_2_, and −OH). Notably, the −OH functionalized TENG device employing the nanocomposite film achieved voltage and current outputs of 197.6 ± 1.3 V and 4.19 ± 0.72 μA, respectively, representing 2.3- and 3.2-fold improvements over the pristine PU film. The underlying mechanism was elucidated through nanoscale chemical and local mechanical characterization, accompanied by further insights via *ab initio* DFT calculations. The results revealed that polar electron-donating groups promote electron localization near the MOF fillers, thereby reducing the overall work function of the MOF@polymer nanocomposite film. The potential practical utility of the optimized tribo-positive TENG was further demonstrated through its integration into a single-electrode shear sensor capable of motion detection, showcasing enhanced shear sensing performance. This study offers valuable insights into MOF-based material design for high-performance TENGs and expands the design space for tunable tribo-positive materials. It paves the way for the development of more sustainable and biocompatible self-powered sensing technologies.

For future deployment of the TENG sensor in wearable and biomedical applications, comprehensive biocompatibility and safety assessments, as well as a systematic evaluation of long-term operational stability, will be essential. Several mitigation strategies may be employed to enhance device robustness against moisture exposure and prolonged mechanical contact. For sensing applications, operation in dry environments or the application of hydrophobic surface coatings may enhance moisture resistance. Alternatively, functional-group engineering of the MOF fillers (e.g., introducing bulky aliphatic substituents) may increase hydrophobicity while preserving their electron-donating characteristics. To address long-term durability, surface lubrication (e.g., with grease) can reduce friction-induced wear. Optimization of the composite membrane thickness and uniform dispersion of filler particles within the polymer matrix can further minimize surface wear and mechanical degradation during repeated operation.

## Supplementary Material



## Notes

Additional experimental and simulation details: photographs of experimental setup and prepared samples (PDF)
