# Supplementary material for: Tuning of Electron-Donating Metal–Organic Frameworks toward High-Performance Triboelectric Nanogenerators for Self-Powered Shear Sensing
Source: ACS Appl Mater Interfaces. 2026 Mar 2;18(12):18221–32. doi: 10.1021/acsami.5c22950 (PMC13051434; doi:10.1021/acsami.5c22950)
Supplement: Supplementary file 1 [file am5c22950_si_001.pdf]

Supplementary Information

Tuning of Electron-Donating Metal-Organic Frameworks towards High Performance Triboelectric Nanogenerators for Self-Powered Shear Sensing

Tianhuai Xu,[1] *Lorenzo Dona*[2]*, and Jin-Chong Tan*[1,*]

[1]Multifunctional Materials & Composites (MMC) Laboratory, Department of Engineering Science, University of Oxford, Parks Road, Oxford OX1 3PJ, U.K.

[2] Department of Chemistry, NIS and INSTM Reference Centre, University of Turin, Torino 10125, Italy.

[*]Corresponding Author: jin-chong.tan@eng.ox.ac.uk

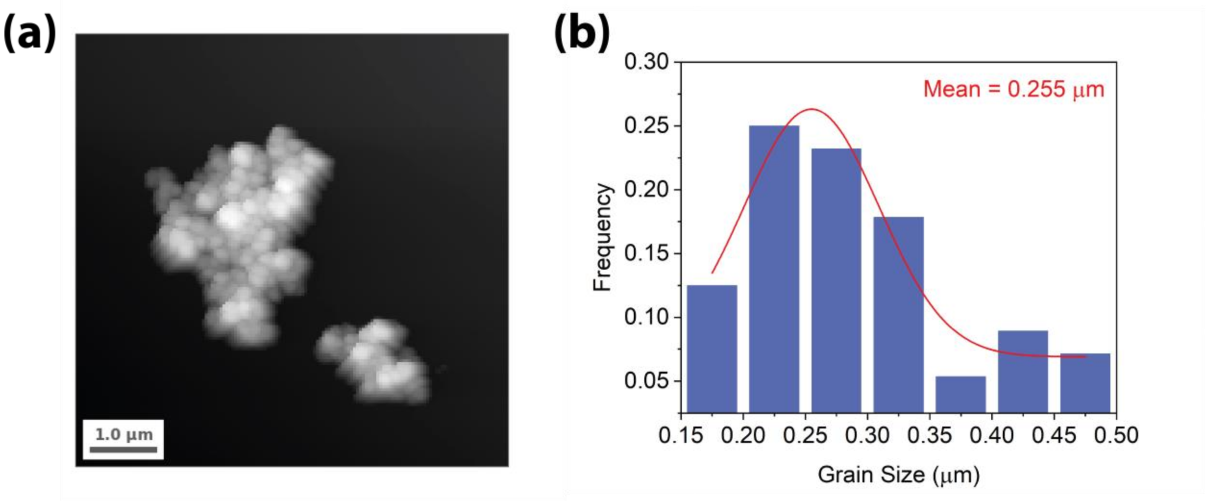

Figure S1. (a) AFM height topography image of the UiO-66-OH crystals. (b) Grain size distribution of the UiO-66-OH crystals derived from nanoparticles imaged in panel (a).

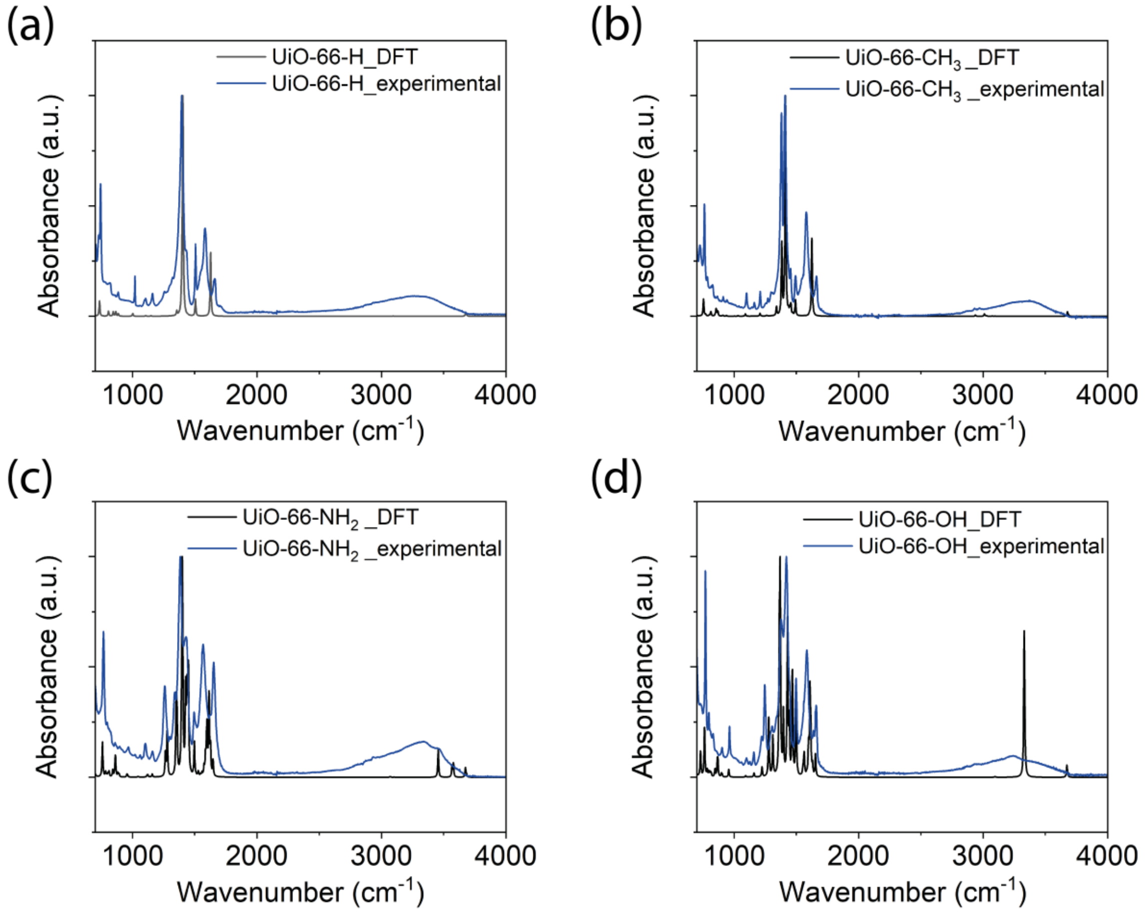

Figure S2. (a-d) Comparison of experimental FTIR results with IR spectra predicted by DFT calculations for all the four UiO-66-X derivatives. A scaling factor of 0.96 was used for the DFT results to account for the anharmonicity effect.

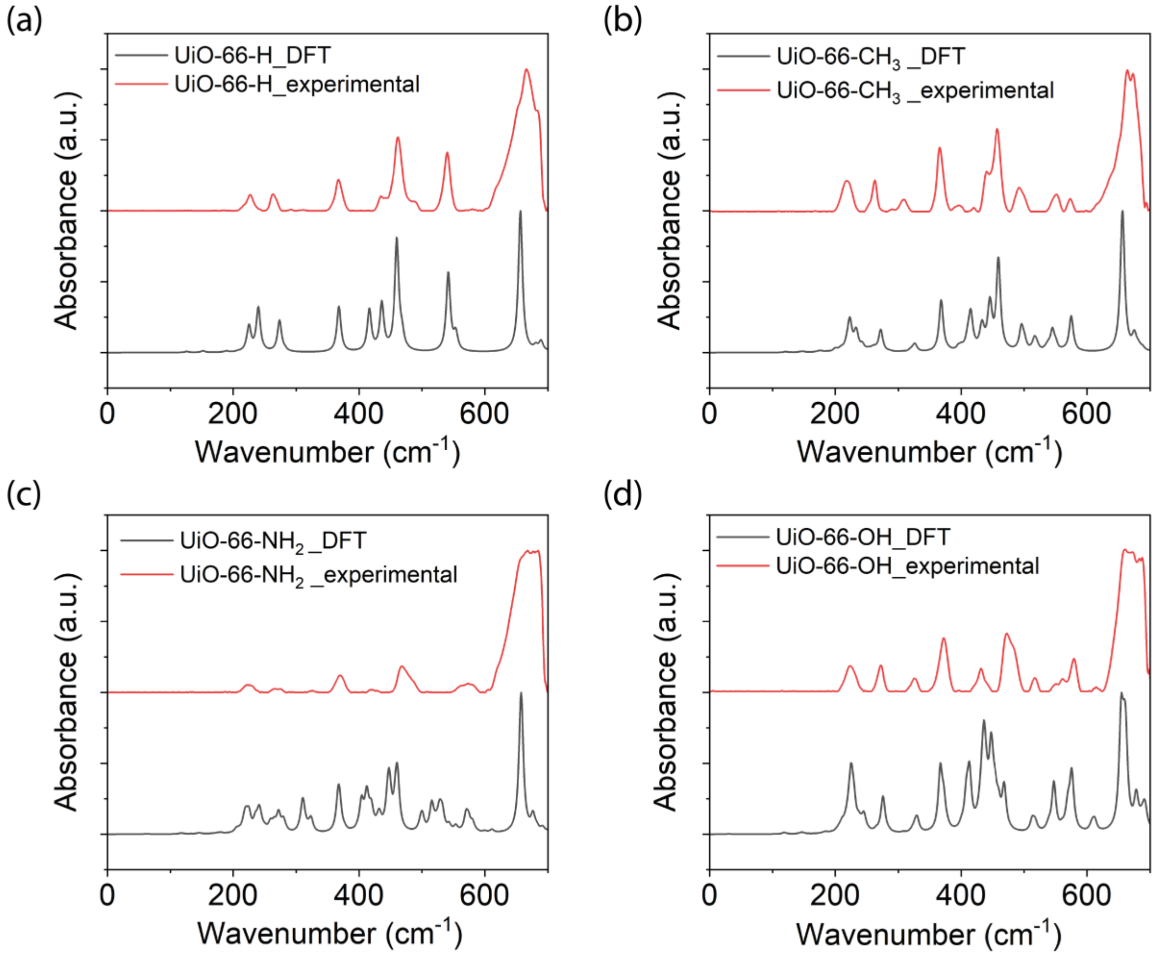

Figure S3. Terahertz modes below ~21 THz. (a-d) Comparison of the synchrotron radiation far-IR spectra with the DFT calculated IR spectra for all UiO-66-X derivatives.

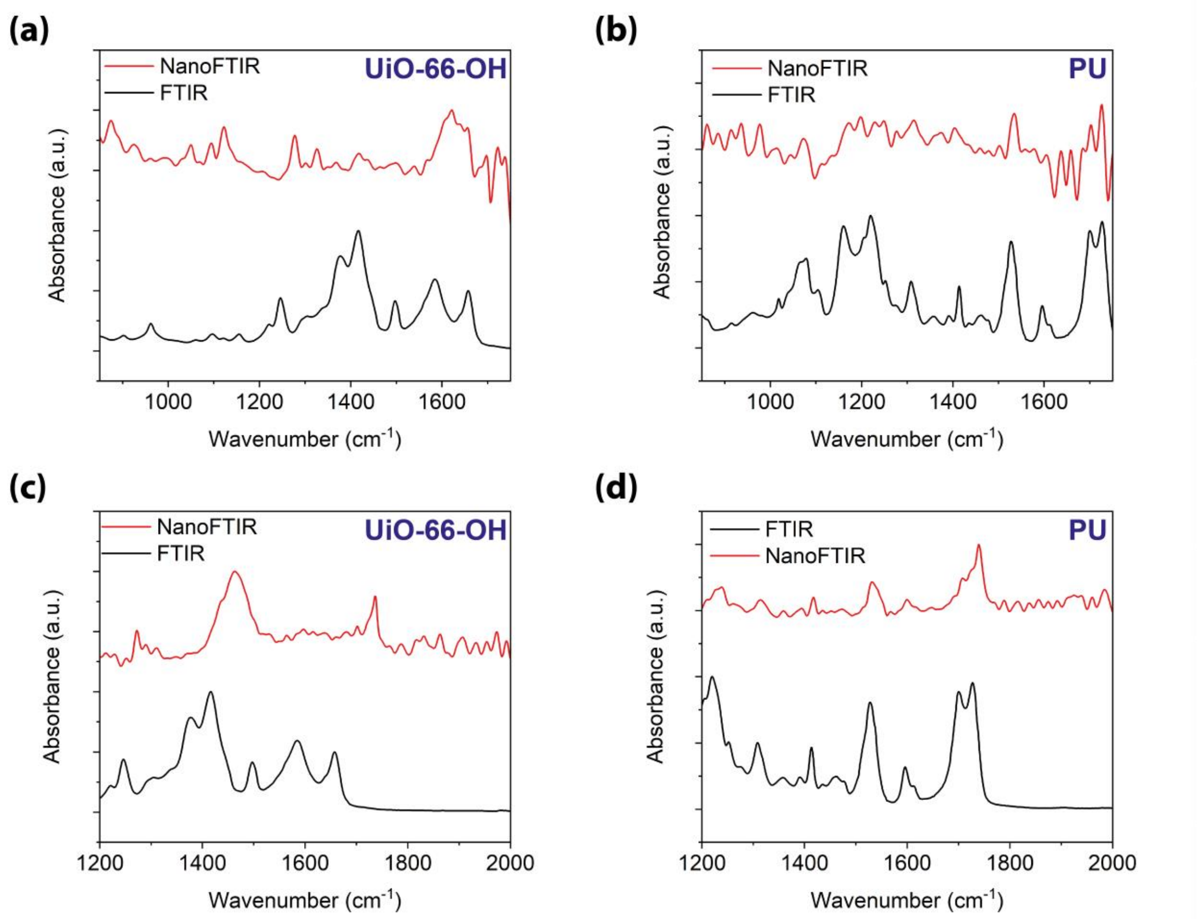

Figure S4. (a-b) Comparison of the (near field) nano-FTIR and (far field) ATR-FTIR bands of the UiO-66-OH and PU from 850 to 1750 cm^{-1}. (c-d) Comparison of the nano-FTIR and ATR-FTIR bands of the UiO-66-OH and PU from 1200 to 2000 cm^{-1}.

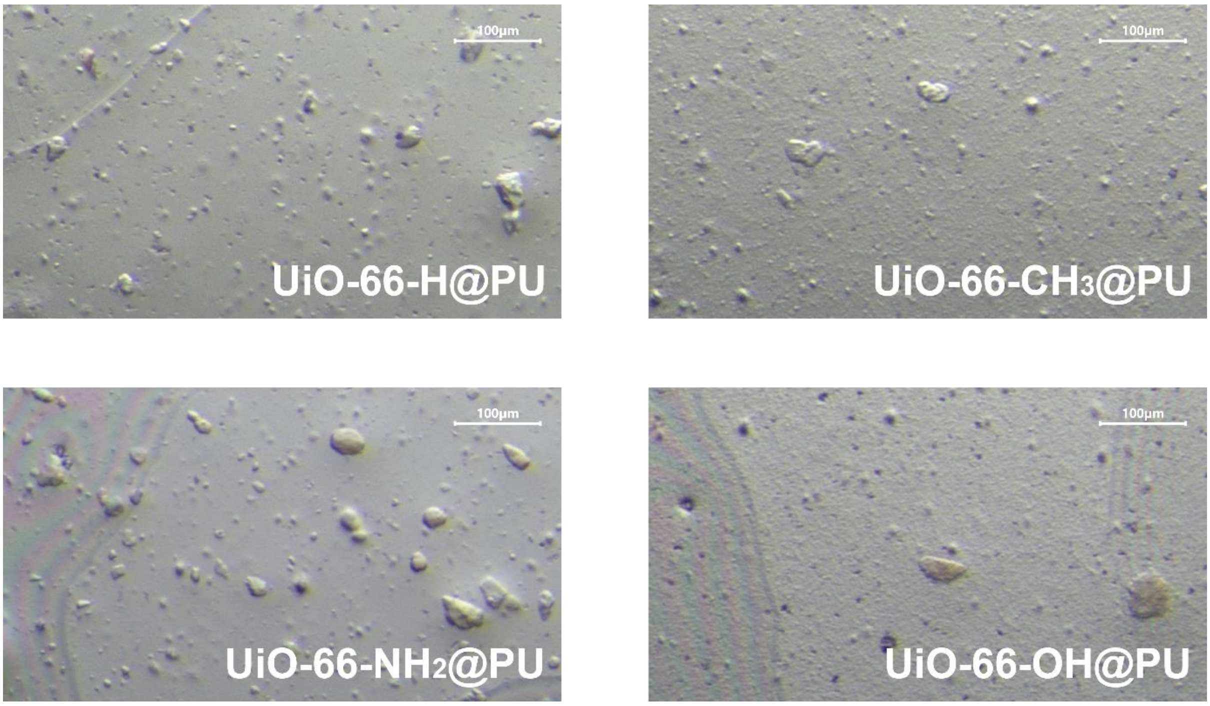

Figure S5. Optical stereomicroscopy of the top surface (with respect to doctor blade) of UiO-66-X@PU composite membranes imaged under the visible light.

(a)

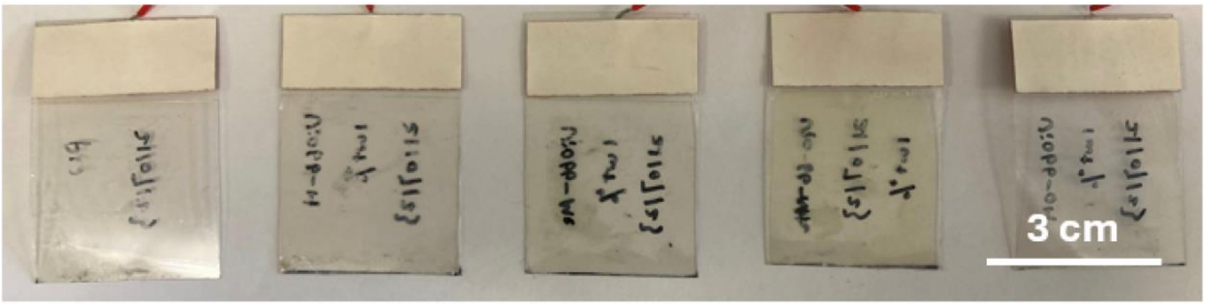

(b)

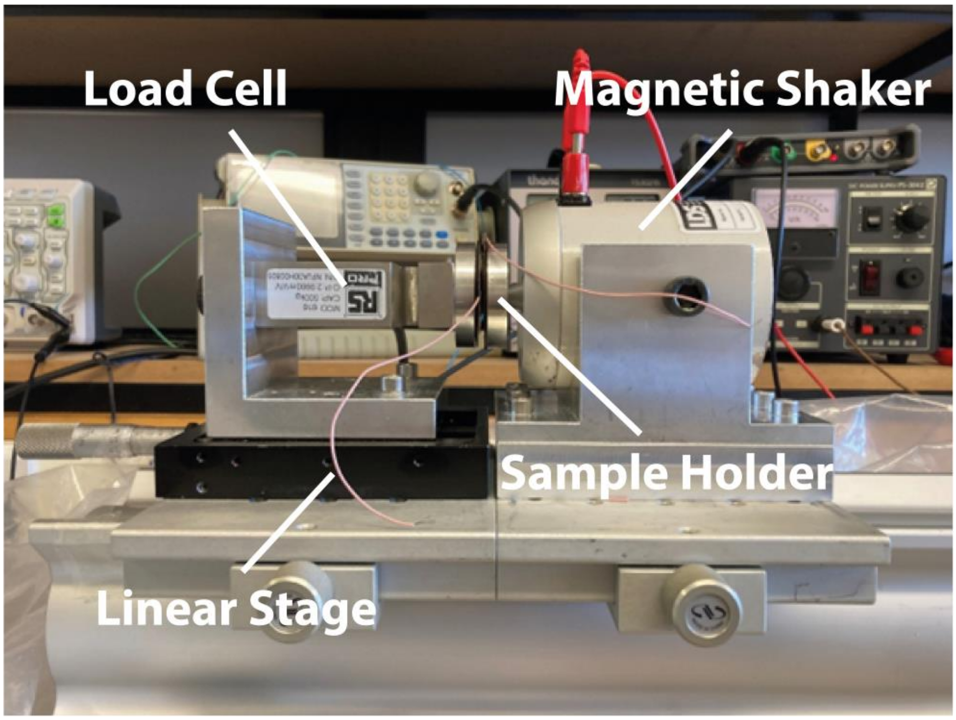

Figure S6. (a) Photographs of the UiO-66-X@PU membrane devices on ITO-coated PET substrates prepared for TENG measurements. (b) Custom built contact-separation mode TENG setup in the MMC Lab.

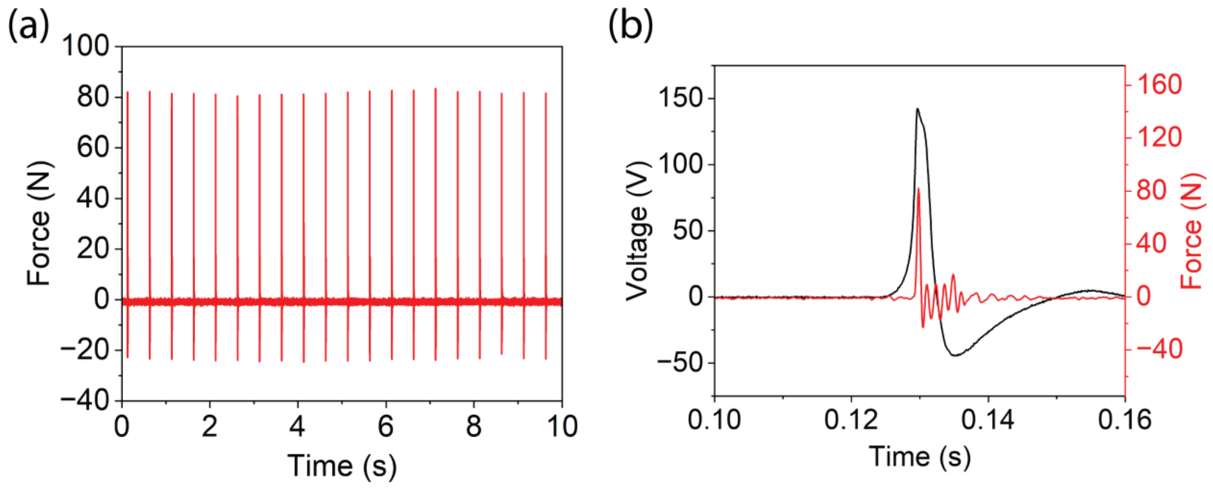

Figure S7. (a) Tapping force during TENG measurements, recorded by the load cell depicted in Figure S6. The maximum instantaneous force is ~80N. (b) A single pulse of the force signal and the corresponding voltage output.

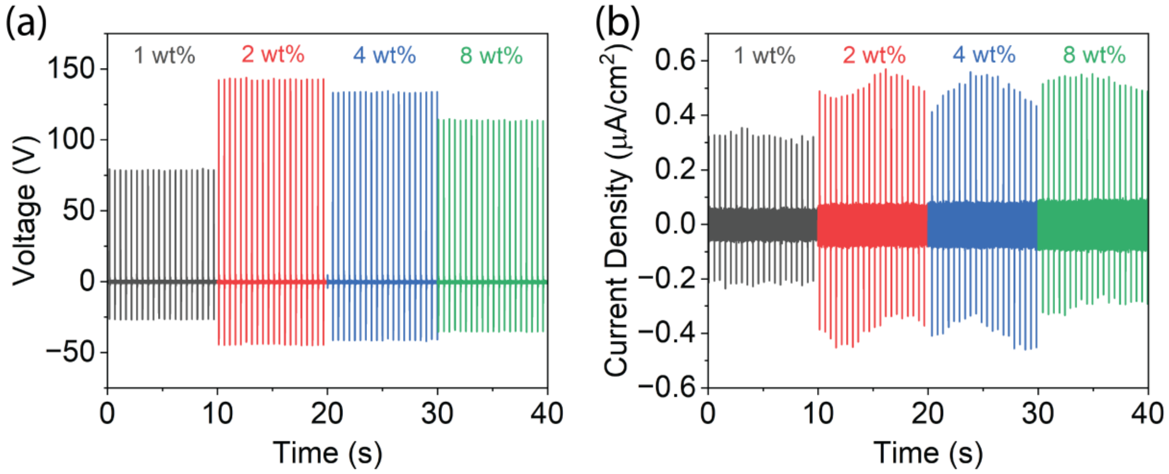

Figure S8. (a-b) Open-circuit voltage and short-circuit current density of the UiO-66-OH@PU TENG devices, employing different weight percent loading of MOF fillers integrated within PU matrix.

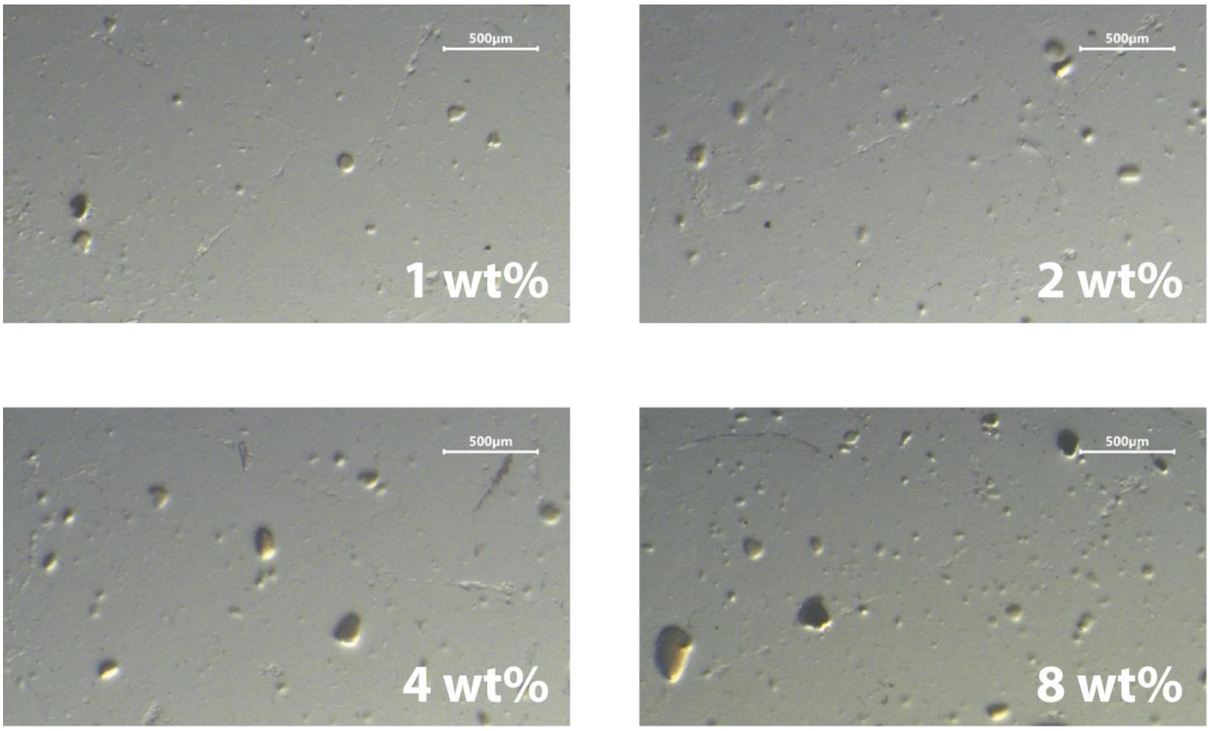

Figure S9. Optical stereomicroscopy of the top surface (with respect to doctor blade) of UiO-66-OH@PU composite membranes with different wt.% loadings imaged under visible light.

Table S1. Comparison of the maximum transferred charge density per unit stress for different UiO-66-X@PU TENGs.

Sample	Maximum charge density per unit stress (μC m^{-2} MPa^{-1})
PU	83.3 ± 3.5
UiO-66-H@PU	121.3 ± 2.5
UiO-66-CH_3@PU	129.0 ± 2.5
UiO-66-NH_2@PU	152.4 ± 4.0
UiO-66-OH@PU	230.5 ± 3.3

Table S2. Comparison of all functionalised UiO-66 based TENGs

Ref	Positive Tribo-Layer	Negative Tribo-Layer	Size	Open-Circuit Voltage (V_{oc})	Short-Circuit Current (I_{sc})	Peak Power Density
1	Cu	UiO-66-NO_2	1 × 1 cm^2	23.8 V	0.29 μA	-
2	UiO-66-NH_2@PVDF	PVDF	5 × 5 cm^2	-	50 μA	1.23 W/m^2
3	Al	UiO-66-4F@PDMS	3 × 3 cm^2	937 V	30.6 μA	38.78 W/m^2
4	Cu	UiO-66-NO_2@PDMS	2 × 2 cm^2	191 V	17.3 μA	-
5	Polyimide (PI)	UiO-66-NH_2@PI	5 × 5 cm^2	~45 V	-	0.78 W/m^2
6	Al	UiO-66-NH_2@PDMS	3 × 3 cm^2	375 V	8 μA	1.69 W/m^2
**This work**	UiO-66-OH@PU	PDMS	3 × 3 cm^2	197.6 V	0.47 μA	0.83 W/m^2

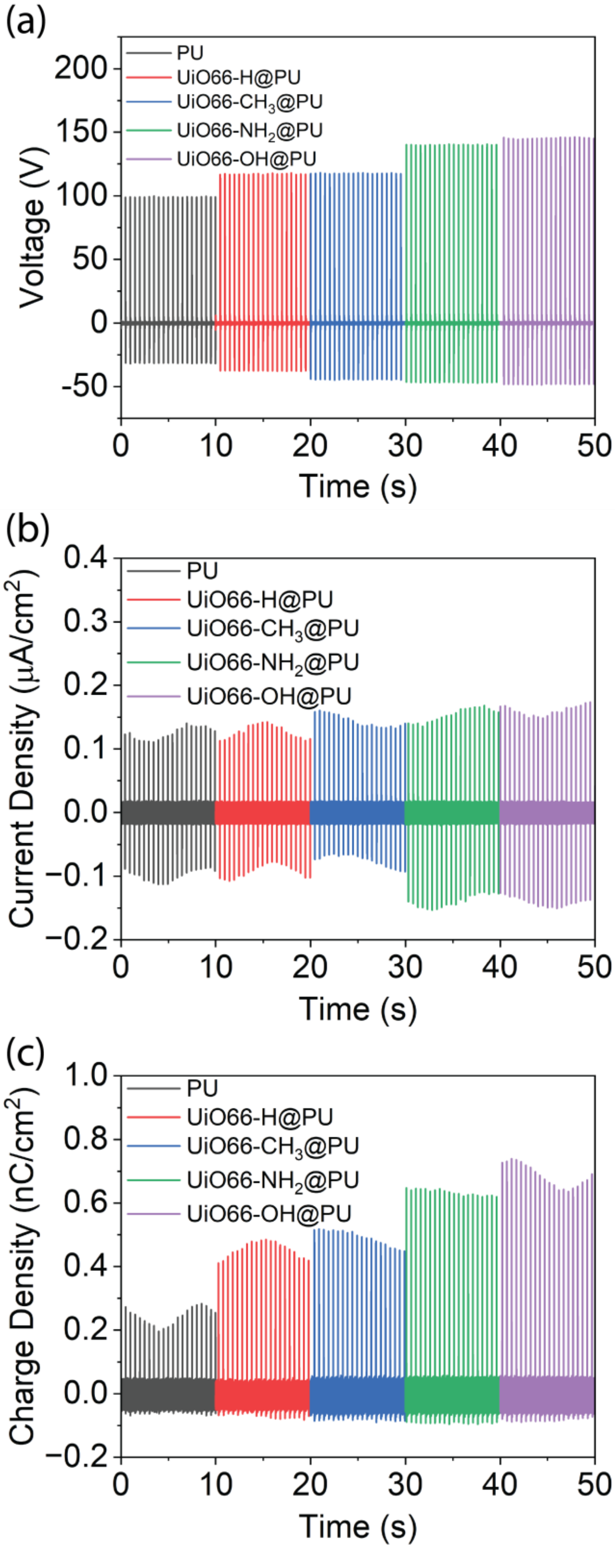

Figure S10. (a) The open-circuit voltage, (b) short-circuit current density, and (c) charge transfer density of another batch of UiO-66-X@PU TENG devices.

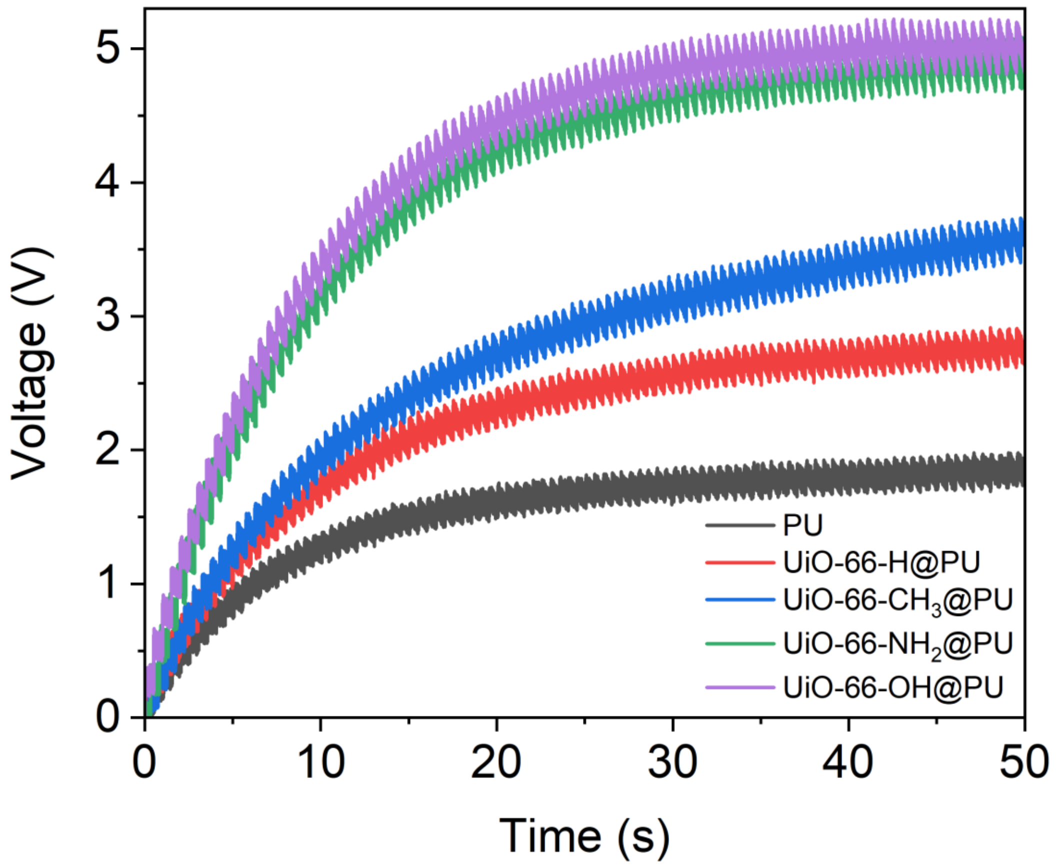

Figure S11. Comparison of the voltage output profiles when charging a 0.1 μF capacitor using different UiO-66-X@PU TENGs over a period of 50 s. Oscillation in voltage reflects the charging cycles due to contact and separation.

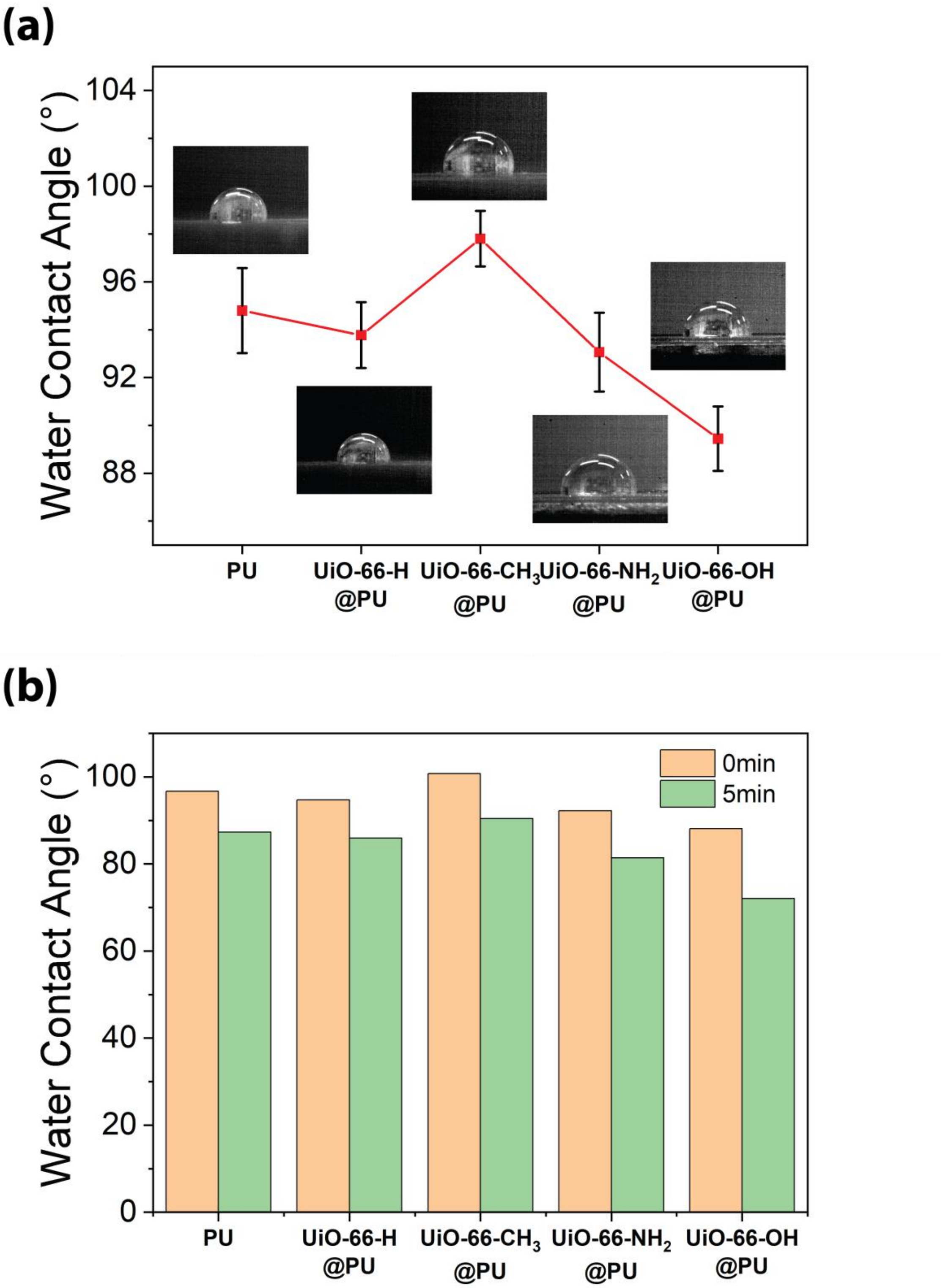

Figure S12. (a) The water contact angles of different UiO-66-X@PU composite films at start of measurement (t = 0 min). Standard deviation derived from 5 measurements. (b)The change in water contact angle before and after 5 minutes.

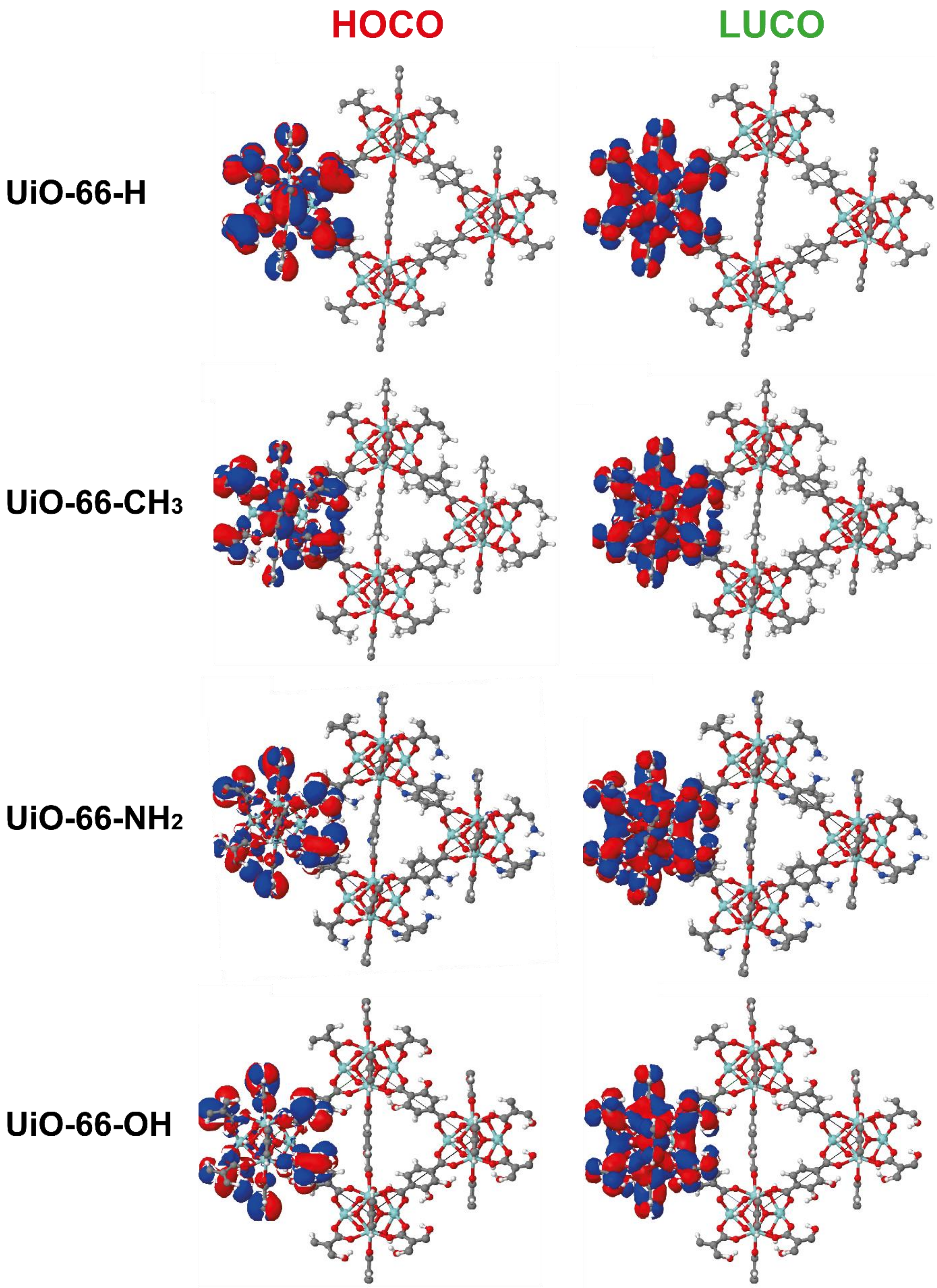

Figure S13. HOCO and LUCO crystal orbitals calculated by DFT for the different UiO-66 derivatives, where the red and blue lobes represent the two phases of the orbital wavefunction.

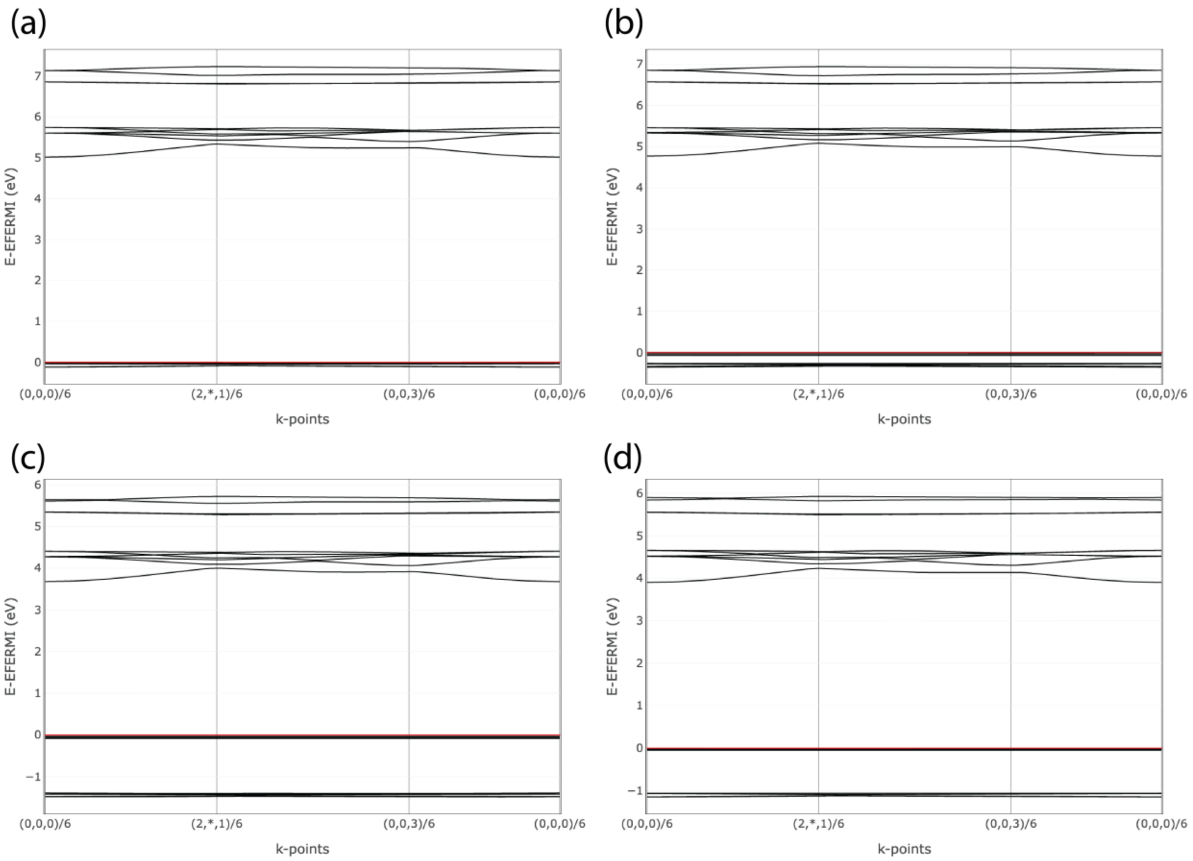

Figure S14. Band-structure plots of (a) UiO-66-H, (b) UiO-66-CH_3, (c) UiO-66-NH_2, and (d) UiO-66-OH calculated at PBEsol0-3c level of theory. Energy values in eV.

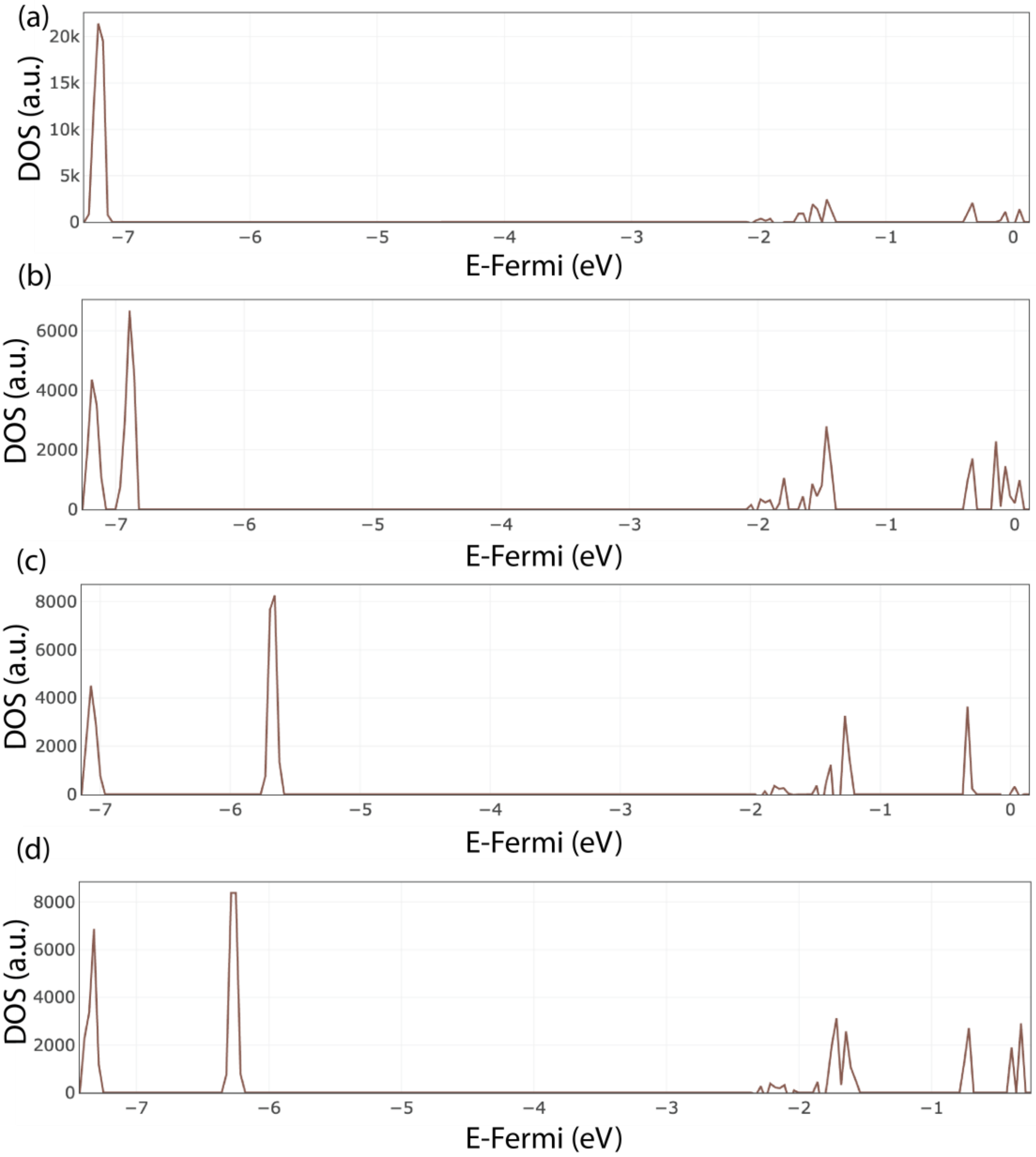

Figure S15. Total density-of-states plots for (a) UiO-66-H, (b) UiO-66-CH_3, (c) UiO-66-NH_2, and (d) UiO-66-OH calculated at PBEsol0-3c level of theory. Energy values in eV.

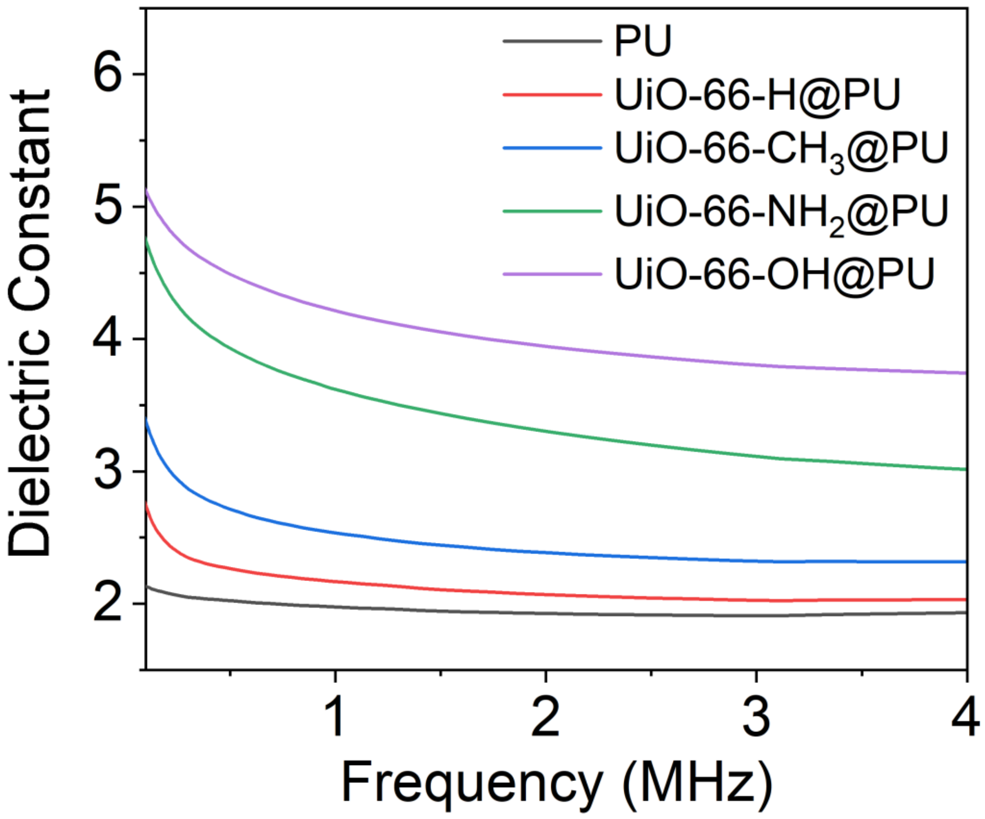

Figure S16. Dielectric spectra of the UiO-66-X@PU composites, measured from 4 Hz to 8 MHz. The dielectric constants were measured using the Hioki-IM3536 LCR meter. The two surfaces of the membrane samples were sputter-coated with a thin layer of conductive metal (Au/Pd), and then the samples were placed in between the two electrodes of the LCR meter for dielectric measurement. The detailed testing procedure can be found in ref[7].

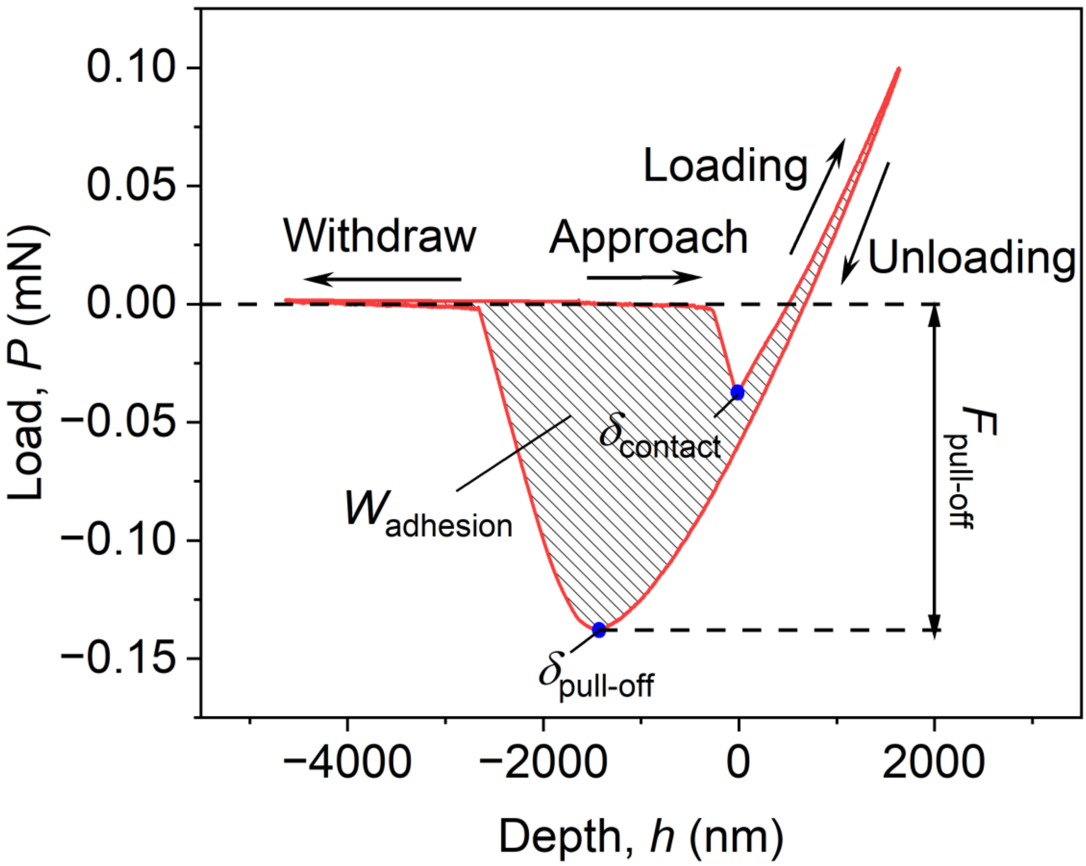

Figure S17. A representative load-depth (*P*-*h*) curve obtained from a pull-off test performed in a nanoindenter equipped with a flat-ended cylindrical probe (radius = 10 μm). During the test, the probe was initially withdrawn 2 μm from the surface to make sure the tip is fully out of the adhesive interaction. The tip then starts approaching the surface at a speed of 100 nm/s until it detects the surface ($\delta_{contact}$), where the sample snaps onto the probe and generates a negative load. After contacting the surface, an indent is performed at a rate of 0.01 mN/s to a maximum load of 0.1 mN. The tip is then held at peak loading position for 2 s, before it starts the unloading process at the same rate. $\delta_{pull\text{-}off}$ and $F_{pull\text{-}off}$ are the indentation depth and the load at the point of maximum adhesive force, respectively. The probe is eventually withdrawn 5 μm from the sample surface. Reproduced with permission from reference 8. Copyright 2024 John Wiley and Sons.

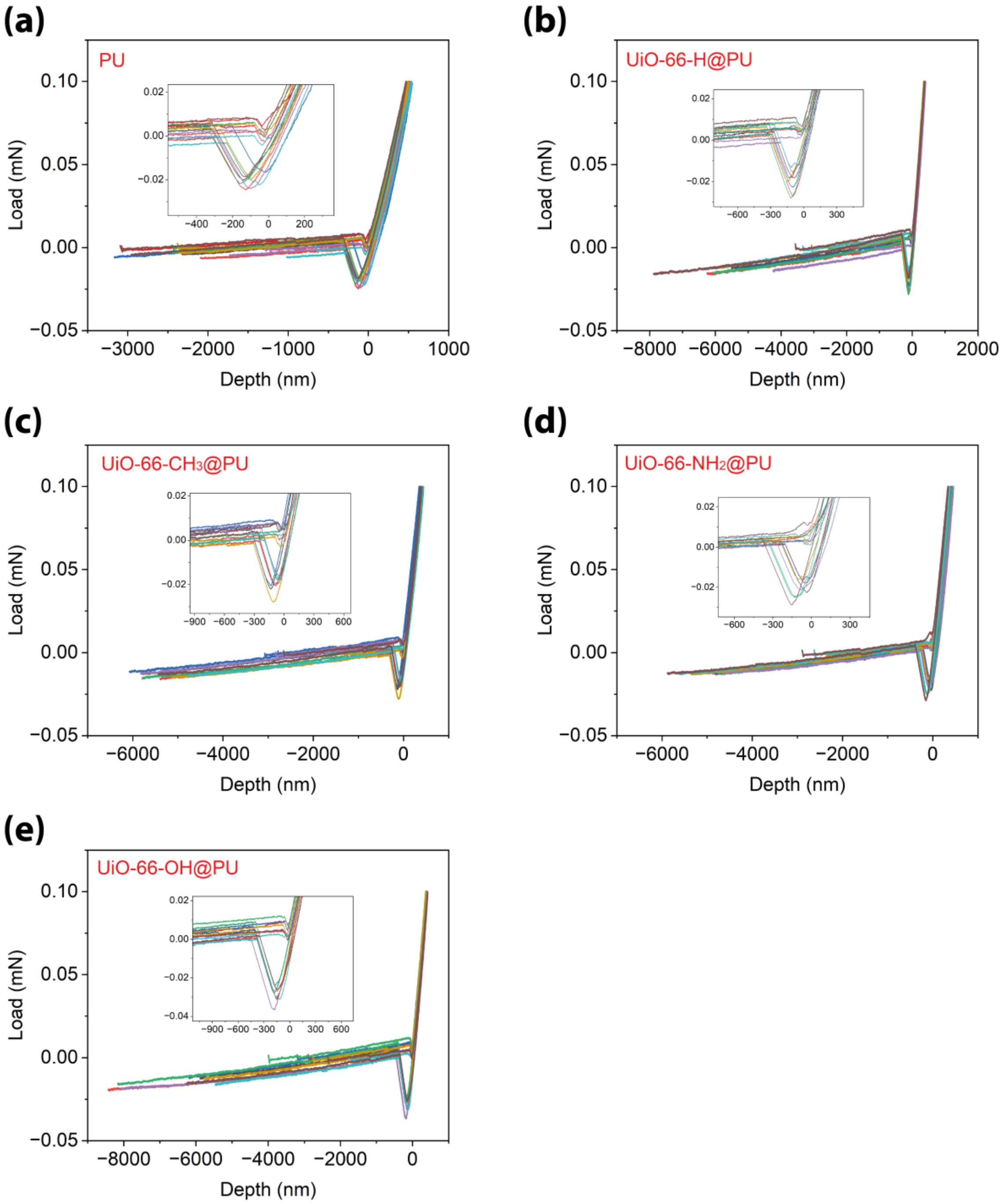

Figure S18. (a-e) Load-depth (*P*−*h*) curves of the pristine PU and UiO-66-X@PU composite films obtained from pull-off surface adhesion tests (see test approach given in Figure S13). Each sample was tested at 8 distinctive positions. The insets show the pull-off regions of each sample.

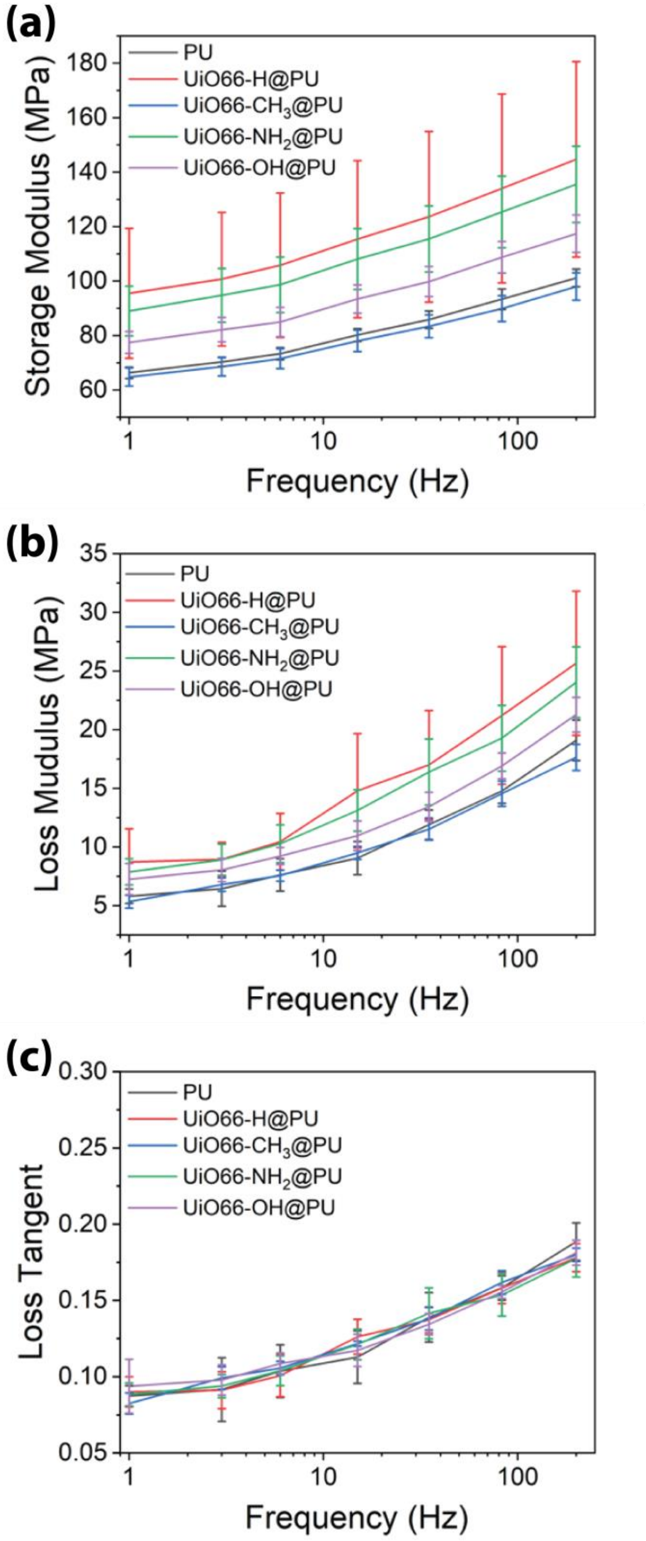

Figure S19. (a-c) Storage modulus, loss modulus and loss tangent of the UiO-66-X@PU composites across different frequencies obtained from probeDMA tests conducted under an iMicro nanoindenter. During the test, a flat punch (radius = 26 μm) was used to apply an oscillating stress on the sample at varying frequencies. The standard deviations were derived from 12 measurements. The detailed testing procedure can be found in ref[8].

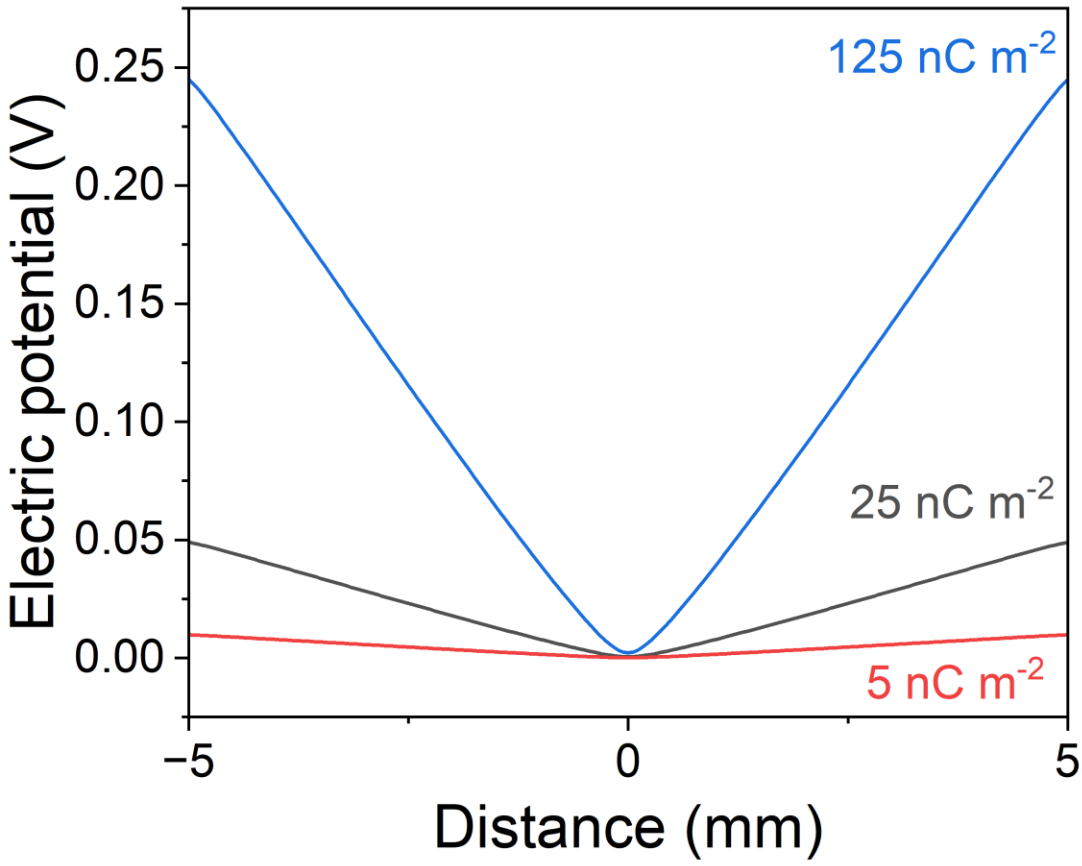

Figure S20. Simulated electric potential profiles at different sliding displacements under different initial surface charge densities.

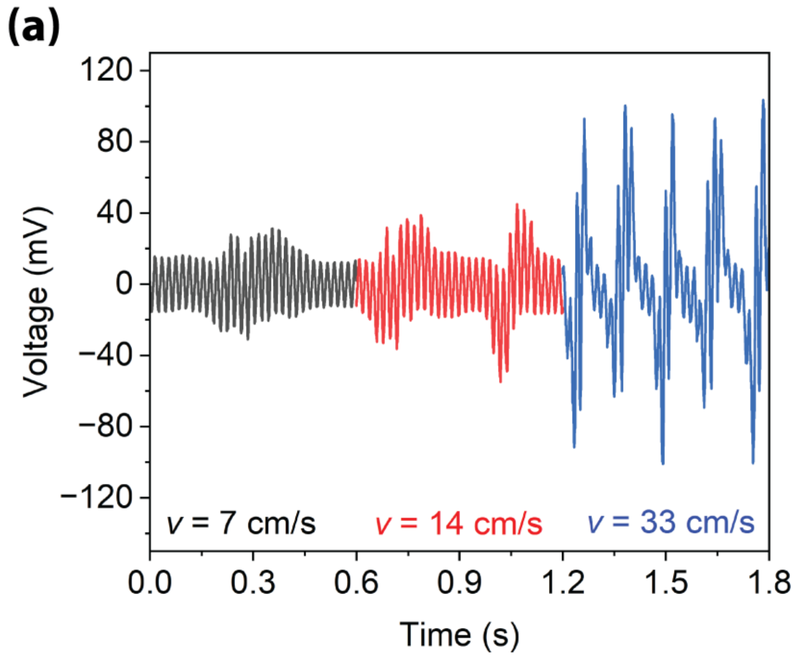

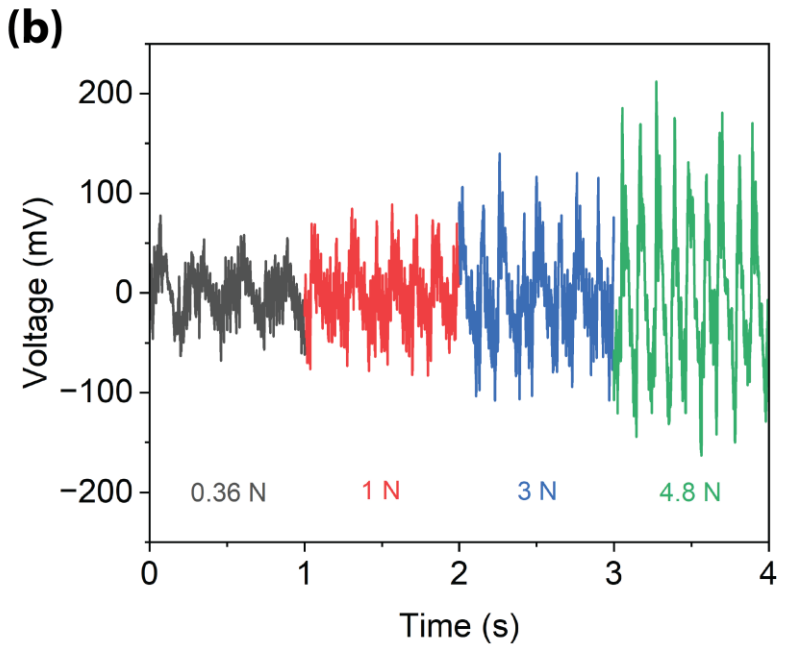

Figure S21. The voltage output of the shear sensor tested under different (a) shear rates and (b) applied normal forces.

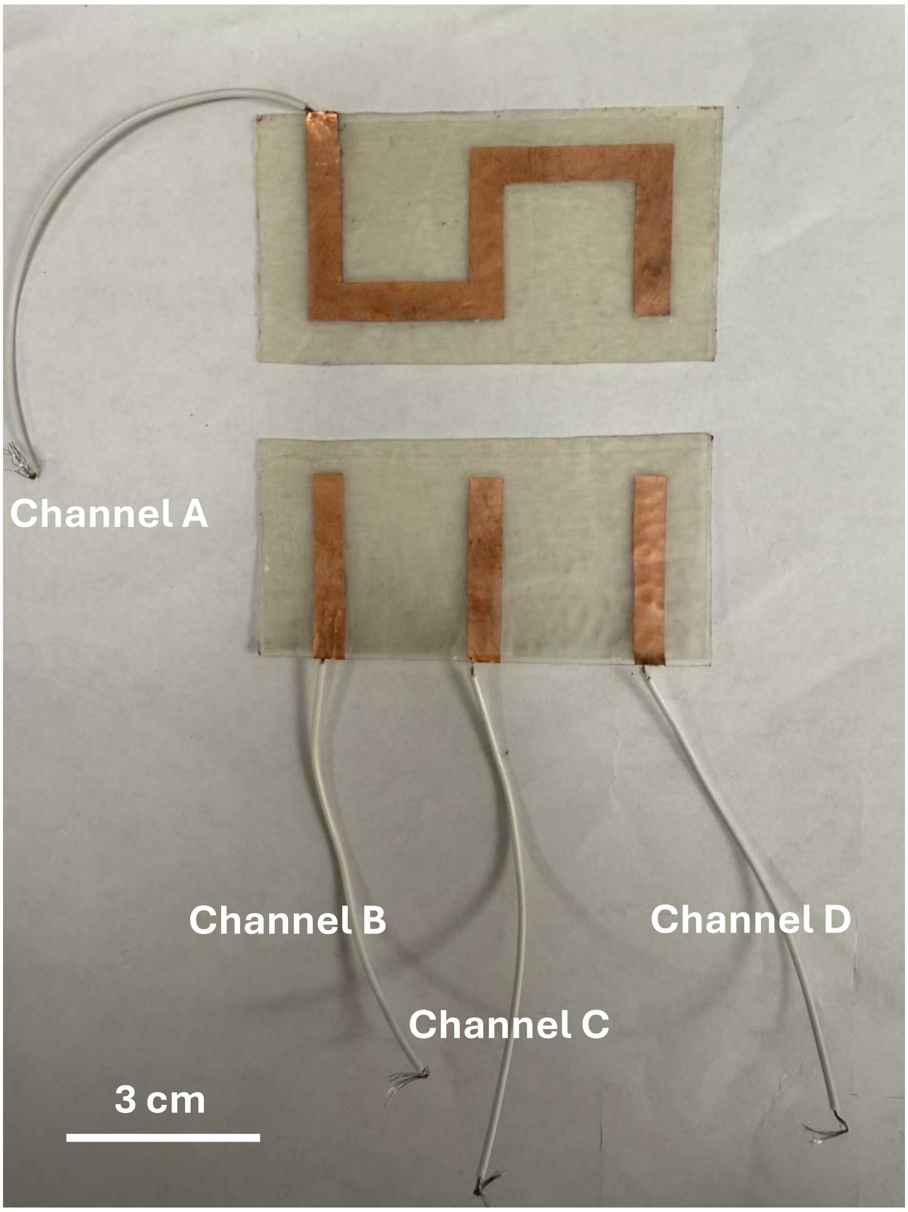

Figure S22. Photograph of the 3-segment TENG-based shear sensors with different electrode configurations (top: 3 electrodes in series; bottom: 3 electrodes in parallel) and the corresponding output channel connected to each electrode.

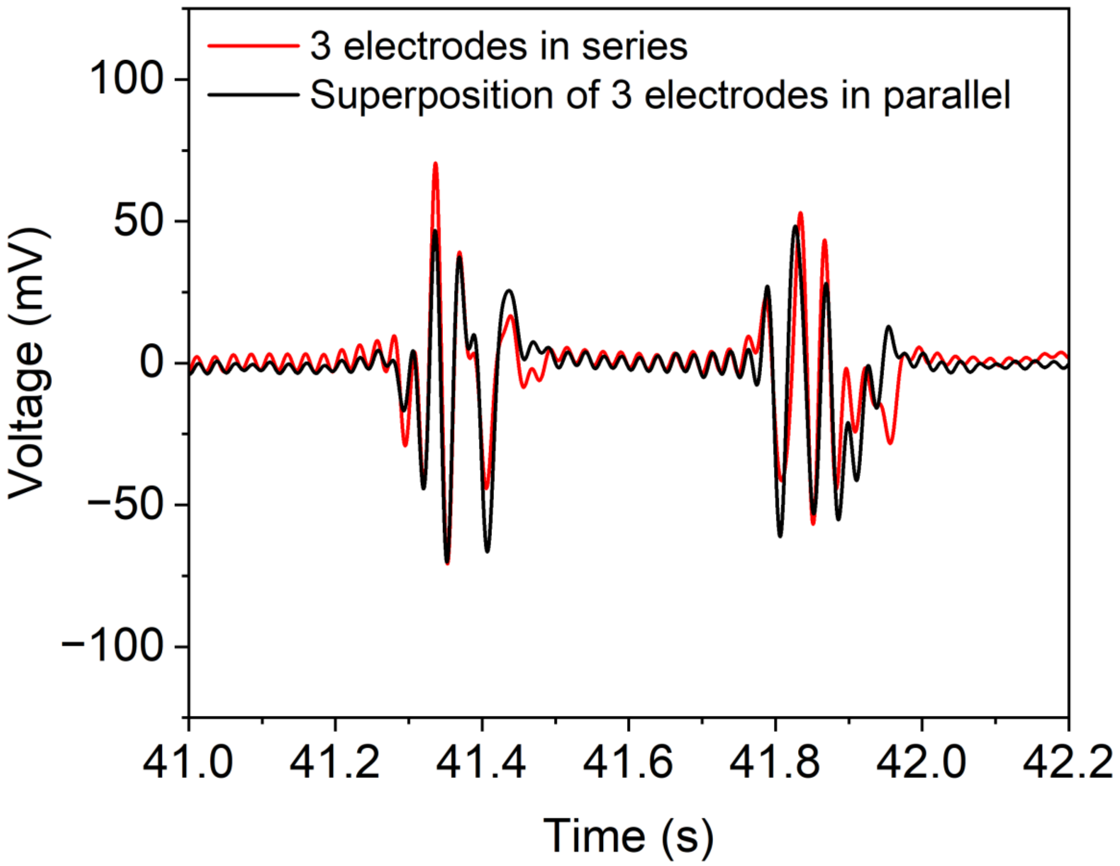

Figure S23. The superimposed signal of the three electrodes in series (Channel A in Figure S15) and the combined signal of the three electrodes in parallel (the sum of Channel B, Channel C and Channel D in Figure S15).

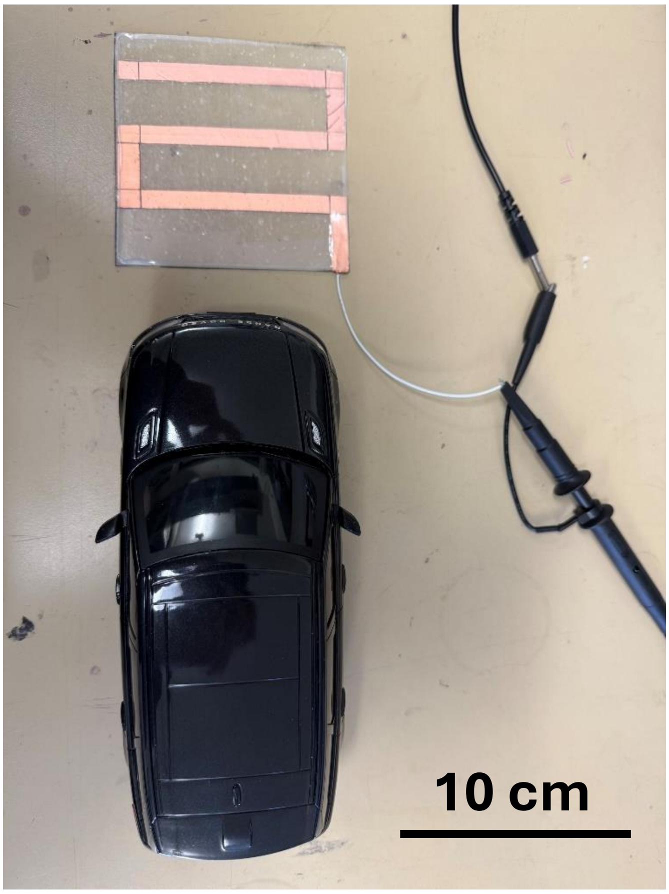

Figure S24. Photo of the large-size shear sensor (dimensions = 10 × 10 cm^2) used for speed detection employing a model car.

References

(1) Wen, R. M.; Feng, R.; Zhao, B.; Song, J. F.; Fan, L. M.; Zhai, J. Y. Controllable Design of High-Efficiency Triboelectric Materials by Functionalized Metal-Organic Frameworks with a Large Electron-Withdrawing Functional Group. *Nano Research* **2022**, 15 (10), 9386-9391.
(2) Zhu, P.; Ullah, Z.; Zheng, S.; Yang, Z.; Yu, S.; Zhu, S.; Liu, L.; He, A.; Wang, C.; Li, Q. Ultrahigh Current Output from Triboelectric Nanogenerators Based on UIO-66 Materials for Electrochemical Cathodic Protection. *Nano Energy* **2023**, 108, 108195.
(3) Wang, Y.-M.; Zhang, X.; Liu, C.; Wu, L.; Zhang, J.; Lei, T.; Wang, Y.; Yin, X.-B.; Yang, R. Remarkable Improvement of MOF-Based Triboelectric Nanogenerators with Strong Electron-Withdrawing Groups. *Nano Energy* **2023**, 107, 108149.
(4) Wen, R.; Zhao, B.; Fan, L.; Guo, J.; Zhai, J. Controlling the Output Performance of Triboelectric Nanogenerator Through Filling Isostructural Metal–Organic Frameworks With Varying Functional Groups. *Adv. Mater. Technol.* **2023**, 8 (6), 2201330.
(5) Wang, J.; Zhou, T.; Zou, J.; Hai, J.; Lu, Y. A High Output Power Density Triboelectric Nanogenerator Based on Surface-Patterned PI/UIO-66-NH_2 Mixed Matrix Membranes. *Nanoscale* **2025**, 17 (48), 28155-28164.
(6) Wang, Y. M.; Zhang, X.; Yang, D.; Wu, L.; Zhang, J.; Lei, T.; Yang, R. Highly Stable Metal-Organic Framework UiO-66-NH_2 for High-Performance Triboelectric Nanogenerators. *Nanotechnology* **2021**, 33 (6), 065402.
(7) Babal, A. S.; Donà, L.; Ryder, M. R.; Titov, K.; Chaudhari, A. K.; Zeng, Z.; Kelley, C. S.; Frogley, M. D.; Cinque, G.; Civalleri, B.; Tan, J.-C. Impact of Pressure and Temperature on the Broadband Dielectric Response of the HKUST-1 Metal–Organic Framework. *J. Phys. Chem. C* **2019**, 123 (48), 29427-29435.
(8) Xu, T.; Ye, J.; Tan, J. C. Unravelling the Ageing Effects of PDMS-Based Triboelectric Nanogenerators. *Adv. Mater. Interfaces* **2024**, 11 (19), 2400094.
